# Interrupted CTG repeats in the 37–43 units size range in the 3ʹUTR of *DMPK* are common alleles

**DOI:** 10.1038/s41431-025-01907-9

**Published:** 2025-07-08

**Authors:** Hilde Swinkels, Maike Leferink, Maartje Pennings, Bart van der Sanden, Christian Gilissen, Jordi Corominas Galbany, Erik-Jan Kamsteeg

**Affiliations:** Department of Human Genetics, Radboud University Medical Center, Nijmegen, the Netherlands

**Keywords:** Neurological disorders, Genetic testing

## Abstract

The size of non-pathogenic CTG repeats in the 3’UTR of the *DMPK* gene varies from 5–35, whereas repeats over 50 units are pathogenic. The Intermediate repeats of 36–50 are considered ‘premutation’, as they are present in individuals unaffected by myotonic dystrophy, but are prone to further enlargement into the pathogenic range upon transmission to offspring. In this study, we showed that CCGCTG interrupted intermediate repeats, in the repeat size of 37–43 units, have been detected in multiple families with a history of myotonic dystrophy. However, segregation and microsatellite marker analysis of these interrupted intermediate alleles revealed that these alleles are not the same alleles (haplotypes) that were found expanded in affected family members. In contrast to the pure intermediate alleles, the CCGCTG intermediate repeats within families did not show intergenerational variability in size. Furthermore, we showed that the CCGCTG interrupted intermediate alleles have an allele frequency of approximately 0.35% in the general population, while CCGCTG interruptions were not detected in pathogenic repeat expansions over 50 repeat units in our control cohort. We postulate that intermediate repeats of size 37–43 having CCGCTG interruptions are not prone to further expansion, and therefore not act as premutations, which has great relevance for individuals with these alleles and has implications for genetic counseling and testing.

## Introduction

Myotonic dystrophy type I (DM1; OMIM#160900) is an autosomal dominant multisystemic disorder characterized by some or multiple of the following features: myotonia, muscular dystrophy, cataracts, cardiac dysfunction, cognitive impairment, daytime sleepiness and fatigue, endocrine involvement, reproductive dysfunctions and pregnancy complications, and frontal balding. The disease shows a high variability in severity and involvement of the different organs. DM1 can be divided into five forms: congenital (prenatal - <1 month), childhood (1–10 y), juvenile (10–20 y), adult (20–40 y) and late adult (onset >40 y) [1–3]. The congenital form of DM1 is the most severe form, presenting at birth or neonatally. Polyhydramnios, decreased fetal movement, and clubfeet can be detected prenatally. Symptoms, which may be life-threatening, are mostly evident at birth and include severe hypotonia, respiratory insufficiency,y and feeding difficulty. Later, cognitive and learning disabilities become apparent. Childhood DM1 is associated with normal development in the first year(s) of life, followed by intellectual deficiency, delayed bowel-bladder training, speech and hearing problems, clumsiness, and cardiac dysrhythmia. Muscle symptoms occur later. Juvenile DM1 is characterized by neurocognitive dysfunction, often manifested as school and behavioral problems, with muscle symptoms appearing later. The adult form is the most variable in system involvement, including skeletal muscle, eye, heart, brain, gastrointestinal tract, reproductive organs, endocrine system, and bone. Additionally, DM1 patients have an increased risk of malignant tumors. Late adult DM1 patients present with combinations of cataracts, baldness, occasionally heart block, mild weakness, and myotonia.

DM1 is one of the most common forms of muscular dystrophy in adults, with an estimated prevalence of ~1 in 11,000 individuals [4]. This disorder is caused by the expansion of an unstable CTG trinucleotide repeat in the 3ʹ untranslated region of the *DMPK* gene [5]. The number of CTG-repeat units in the *DMPK* gene correlates with the severity of the disease and the age of onset. In subsequent generations, increasing phenotype severity and decreasing age of onset can occur, which is virtually always associated with an increase in CTG repeat length upon transmission. This phenomenon is called anticipation [2]. The number of CTG-repeat units in individuals not affected by DM1 ranges from 5 to 35 [6]. These non-pathogenic alleles are usually stable upon transmission. *DMPK* alleles containing CTG-repeat units in the range of 36 and 50, identified in unaffected individuals, have been shown to demonstrate an increased risk of instability on transmission. These intermediate alleles are thus considered ‘premutation’ alleles that confer a risk of further expansion into the pathogenic range in offspring [6, 7]. In general, mildly affected patients have 51–150 repeat units and more severely affected patients have *DMPK* alleles with sizes >150 CTG-repeat units. Patients with congenital onset can have repeat expansions of more than 2000 repeat units [6, 8, 9]. There is, however, overlap in repeat lengths within the clinical forms of DM1 [10].

Several studies also proposed that the presence of interrupted pathogenic repeat expansions are associated with altered repeat stability and/or atypical disease phenotypes, suggesting that the presence of interruptions protects the repeat from expanding in further generations [11, 12]. Interrupted repeats are found in approximately 3–5% of the DM1 population [8, 9].

The cut-off value of 35 repeats separating non-pathogenic from intermediate alleles is based on the analysis of >2900 chromosomes of unaffected family members of DM1 families [7]. Occasionally, alleles of the intermediate size (36–50 repeats), but with CCGCTG interruptions, were detected in families where an independent large CTG repeat in *DMPK* was segregating with myotonic dystrophy [8, 9]. Additionally, these interrupted intermediate alleles were transmitted without changes in repeat number. Similarly, a CCGCTG interrupted allele of 37 repeats was shown to be much more stable in sperm than pure repeats of similar size [13].

In this study, we examined the presence of interruptions, and segregation of intermediate *DMPK* alleles (36–50 repeat units) from 17 unrelated DM1 families from a diagnostic cohort. Next, we determined the frequency of interrupted intermediate alleles in the general population from exome sequencing data. We showed that intermediate alleles with a CCGCTG interruption do not have an increased risk of further expansion in subsequent generations, which has major implications for genetic testing and counseling.

## Material and methods

### Patients and control groups

For this study, two cohorts were used. The first cohort is a cohort of 3032 patients specifically tested for DM1 between January 2010 and May 2020 in the Radboudumc in a diagnostic setting. Individuals with repeat sizes of 35–50 units were selected from this cohort. Once identified, datasets of these individuals and their family members, and their corresponding samples, were stripped from personal identifications (de-identified), though information regarding the family relationships were kept for the analyses. The second cohort is an anonymized exome sequencing (ES) cohort consisting of 41,113 samples from trio-based or singleton sequencing. This ES cohort was used to assess the frequency of interrupted intermediate *DMPK* alleles by CCGCTG hexamers in the general population.

### PCR and Fragment length analysis

The number of CTG units was determined for each sample using synthetic fluorescently-labeled primers (Forward: 5ʹFAM-CTCGAAGGGTCCTTGTAGCC-3ʹ and Reverse: 5ʹ-TGCACAAGAAAGCTTTGCAC-3ʹ) flanking the CTG-repeat region. A reaction volume of 20 µl was used containing 100 ng of genomic DNA. The PCR amplification conditions comprised an initial denaturation of 94 °C for 10 min then 30 cycles of denaturation at 95 °C for 30 s, annealing at 60 °C for 30 s with extension at 72 °C for 1 min and a final extension at 72 °C for 7 min. This was followed by direct analysis of the length of the amplified products by capillary electrophoresis using the LifeTechnologies 3730 XL Analyzer (Thermo Fisher). The analysis of results was performed using Genemarker V2.6.7 (SoftGenetics LLC).

### Triplet-repeat primed (TP)-PCR

The TP-PCR was performed using two gene specific primers that lie outside the repeat (P1: 5ʹFAM-GAAGGGTCCTTGTAGCCGGGAA-3ʹ and P3: 5ʹ-TACGCATCCCAGTTTGAGACG-3ʹ) and a third primer designed across the repeated sequence (P4: 5ʹ-TACGCATCCCAGTTTGAGACGCAGCAGCAGCAGCAG-3ʹ). A reaction volume of 25 µl was used containing 100 ng of genomic DNA. The following cycling conditions were used: 1 cycle of 95 °C for 2 min, and 36 cycles of 95 °C for 30 s, 65 °C for 30 s and 70 °C for 1 minute, followed by 1 cycle of 72 °C for 5 min. The TP-PCR results in fragments of different sizes, which have been analyzed by capillary electrophoresis.

### Sequencing of the repeat region

Standard PCR amplification of the repeat region was performed using primers flanking the *DMPK* CTG repeat (Fw- 5ʹ-CCGTTGGAAGACTGAGTGC-3ʹ and Rv- 5ʹ-CTGGCCGAAAGAAAGAAATG-3ʹ). After amplification, the quality of the PCR products was tested using the 4200 TapeStation System (Agilent, Santa Clara, California, USA) and quantified using the Qubit 4 fluorometer (Invitrogen, Thermo Fisher Scientific, Waltham Massachusetts, USA) for normalisation. PCR products were purified using the SMRTbell clean-up procedure and SMRTbell libraries were prepared using the SMRTbell Template Prep Kit 3.0 (Pacific Biosciences, Menlo Park, California, USA). Binding was performed using the Sequel II Binding Kit 3.1 (Pacific Biosciences, Menlo Park, California, USA) and sequencing conditions included 1 h pre-extension and 10 h movie time per SMRT cell. Single molecule circular consensus sequences were generated and analyzed using Integrative Genomics Viewer (Version 2.15.4).

Sanger sequencing was also used to examine the repeat region. After amplification, the PCR products were purified using Multiscreen PCR Cleanup kit (Millipore, Molsheim, France) and directly sequenced on a LifeTechnologies 3730 XL Analyzer (Thermo Fisher Scientific, Waltham Massachusetts, USA).

### Genetic markers

Segregation analysis has been performed using genetic markers to define alleles. Six polymorphic microsatellite markers surrounding the *DMPK* gene (D19S217, D19S574, D19S918, D19S219, D19S112, and D19S902) have been tested using fragment length analysis. Fragment length analysis was performed using a LifeTechnologies 3730 XL Analyzer (Thermo Fisher Scientific, Waltham Massachusetts, USA).

### Occurrence of CCGCTG repeat variant in the general population

The STR detection tool ExpansionHunter was used to extract samples containing the CCGCTG interruption from the ES cohort. The standard CAG (reverse sequence) pattern for the *DMPK* STR has been modified to the pattern of the interruption: CGGCAG by adapting the JSON file (supplementary method file). The entire ES cohort was analyzed using this modified pattern. The ES data consisted of Agilent SureSelect Human All Exon 50 Mb Kit (Santa Clara, CA, USA) enrichment and sequencing on an Illumina Hiseq 2000 or 4000 machine (San Diego, CA, USA). All samples with a possible interruption, and coverage of 10 or higher ( ~ 96% data) were then manually curated by two researchers. The manual curation consisted of viewing ExpansionHunter output files visualised by GraphAlignmentViewer (https://github.com/Illumina/GraphAlignmentViewer). This graph shows the aligned reads containing the repeat region with the corresponding flanking region. In case the two researchers agreed on the presence or absence of the interruptions, as well as on the size of the repeat, the result was passed. If there was no consensus between the two researchers, or if results were uncertain, the samples were validated by Sanger sequencing (20 samples).

## Results

### Intermediate *DMPK* alleles in unaffected family members from myotonic dystrophy patients are often interrupted by CCGCTG’s

**Fig. 1:**
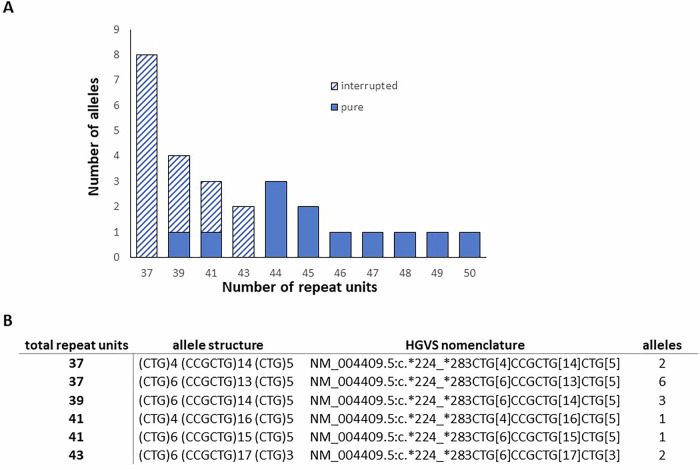
Distribution and structure of intermediate DMPK alleles from a diagnostic cohort. For each individual, only the intermediate allele size is shown. **A** The patterned bars indicate alleles having an interrupted repeat while the solid bars indicate pure CTG repeat. The number of alleles and triplet repeat size are indicated. **B** All interrupted repeats are of the same structure (CTG)n(CCGCTG)n(CTG)n, but differ in the number (n) of pure CTG stretched surrounding the interruption that itself also varies in its length (*allele structure*). The differences in the allele structures are reflected in the total length of the ‘triplet’ repeat (***total repeat units***). The number of occurrences of each allele structure in the cohort is indicated (***alleles***). The alleles are present in the LOVD database under id 1030179, 1030181, 1030182, 1030183, 1030184, 1030185 (from top to bottom).

Our diagnostic cohort consisted of 3032 individuals tested for the *DMPK* repeat by means of locus-spanning PCR and repeat-primed PCR, followed by fragment length analysis. Twenty-seven individuals from 17 different families of this cohort had an intermediate repeat, defined as 36–50 repeat units. These individuals were unaffected and had predictive testing (*n* = 21) or had a different phenotype but were tested because of the familial variant and the notion that myotonic dystrophy is a multisystemic disorder with sometimes nonspecific presentations. Repeat sizes in this group varied from 37-50, though there was a bias towards the shorter allele of 37 units (Fig. 1A).

In some individuals with intermediate alleles, triplet-primed-PCR showed allelic dropout or an unusual pattern with a gap (Supplementary Fig. 1). This indicates the presence of non-CTG repeat units prohibiting binding of the primers. Subsequent sequencing of PCR fragments using PacBio Sequel IIe revealed that 15 of 27 individuals with an intermediate allele size had an interrupted repeat (Fig. 1A). These interruptions were found in repeats with a total size of 37–43 units, whereas no interruptions were seen in the group of 44–50 repeat units. In all cases the interrupted repeat had multiple alternating CCG units flanked by normal CTGs, (CTG)n(CCGCTG)n(CTG)n, (Fig. 1B).

### CCGCTG interrupted intermediate *DMPK* alleles and expanded alleles within the same families do not share the same haplotype

In the families under study, it was initially assumed that the intermediate alleles and the pathogenic expanded alleles in the *DMPK* gene originate from the same ancestor. However, finding intermediate alleles with interruptions that were not present in the expanded pathogenic alleles in the same family, prompted us to perform segregation analyses. Where needed, genetic markers were used to determine haplotypes. Fourteen families could be assessed for these segregation studies.

**Fig. 2:**
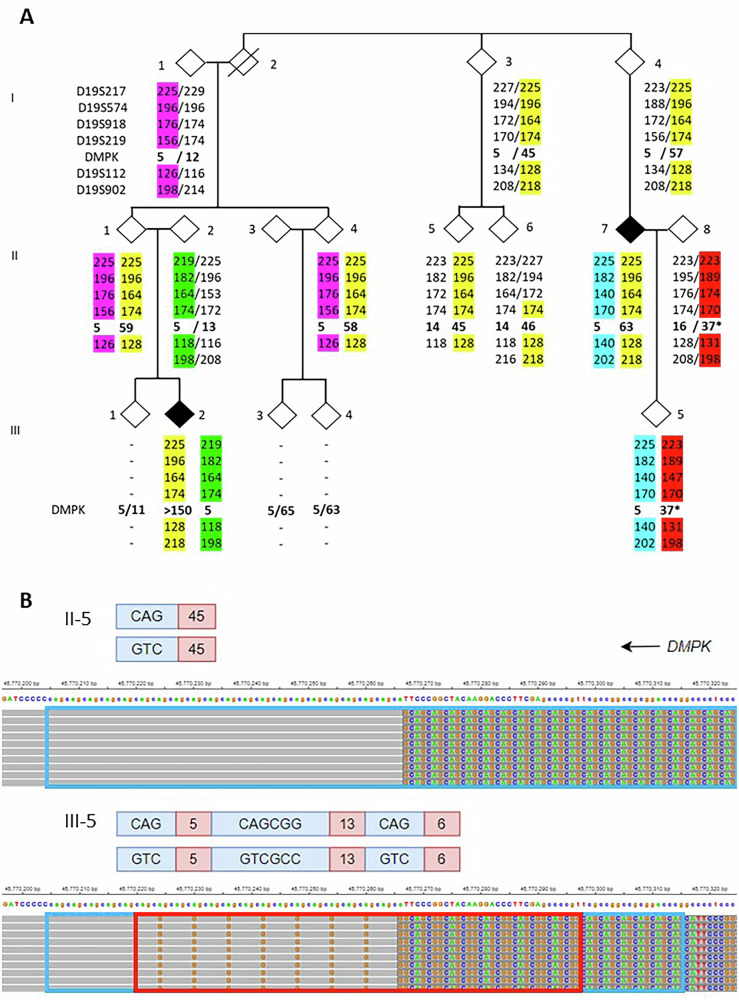
A DM1 family with expanded (pathogenic) and intermediate alleles. **A** Pedigree of *DMPK* family A with three generations (**I-III**), showing unaffected (open symbols) and affected individuals (solid symbols). Males and females are both indicated by diamonds and their position (left and right) within couples is random. The *DMPK* repeat length (*DMPK*) of each individual tested is shown in bold below the symbols, aligned with the sizes of genetic markers surrounding the *DMPK* locus (marker names shown at individual I-1). Different haplotypes are marked with unique background colors. Interrupted *DMPK* alleles are indicated by an asterisk. (B) Partial BAM file of PacBio sequencing data of the 45 repeat allele of individual II-5 and the 37 repeat allele of individual III-5 (both indicated). The sequence data was aligned to the *DMPK* gene (note the orientation of the gene on the minus strand) and bases are only shown when mismatching the reference sequence. The partial BAM file of the 37 repeat allele of individual III-5 is divided into three parts, where the CAG repeat units are surrounded by a blue frame and the CAGCCG repeat units by a red frame. On top of each BAM file alignment a schematic representation shows the repeat structure of both complementary strands with the sequence in blue and the number of triplets in red.

Four of these families had members with CCGCTG-interrupted intermediate alleles that, without exceptions, were shown to have a different origin than the expanding pathogenic alleles present in affected family members (Supplementary Fig. 2). By contrast, all individuals with pure (uninterrupted) intermediate alleles in eight families shared the same haplotype as the affected individuals with expanded alleles (Supplementary Fig. 3). In two families, both interrupted and uninterrupted alleles were present (Fig. 2 and Supplementary Fig. 4). Family A serves as an example, where the interrupted intermediate allele (Fig. 2A; red haplotype; Fig. 2B sequence in lower panel) in individual III-5 was inherited from her unaffected parent, while her affected parent (II-7) has an expanded allele in a different background based on the genetic markers (yellow haplotype). Note that I-3, II-5 and II-6 have pure intermediate alleles (sequence of II-5 in upper panel Fig. 2B) belonging to the same haplotype (yellow) as individuals I-4, II-1, II-4, II-7 and III-2. In contrast to the pure repeats that showed intergenerational instability, all interrupted alleles were transmitted without a change in length (Fig. 2A, II-8 to III-5; supplementary Figs. 2–4 and supplementary table).

These findings reveal that CCGCTG interrupted intermediate alleles are independent occurrences in families with myotonic dystrophy type 1, and suggests that these interrupted alleles are more stable upon transmission to offspring than pure repeats of similar size.

### CCGCTG interrupted *DMPK* alleles occur in ~ 0.35% of the general population

To determine the presence of CCGCTG interrupted alleles in the general population, we analysed a large cohort of exome sequencing datasets (41,113 samples) with ExpansionHunter as described before [14]. This cohort contains both unaffected parents used in trio-exome sequencing and patients tested for disorders other than myotonic dystrophy (both in trio sequencing as singletons). To search for interrupted *DMPK* alleles, we adapted the search to identify CCGCTG hexamers at the *DMPK* locus. To avoid artefacts a threshold was set for each sample to have at least two CCGCTG interruptions and the *DMPK* locus had to be covered at least 10 times. With this, 442 samples with a potential interruption of more than one CCGCTG repeat were extracted from the exome cohort. Manual curation of all 442 samples by identifying the interruption in the *DMPK* graph images, and Sanger / Sequel IIe sequencing validation of equivocal samples (data not shown), led to the identification of 301 samples with a bona fide CCGCTG interruption on one allele. All aberrant alleles had stretches of at least eight of these interruptions. Thus, ~0.7% of the entire cohort had one CCGCTG-interrupted allele (allele frequency of ~0.35%).

**Fig. 3:**
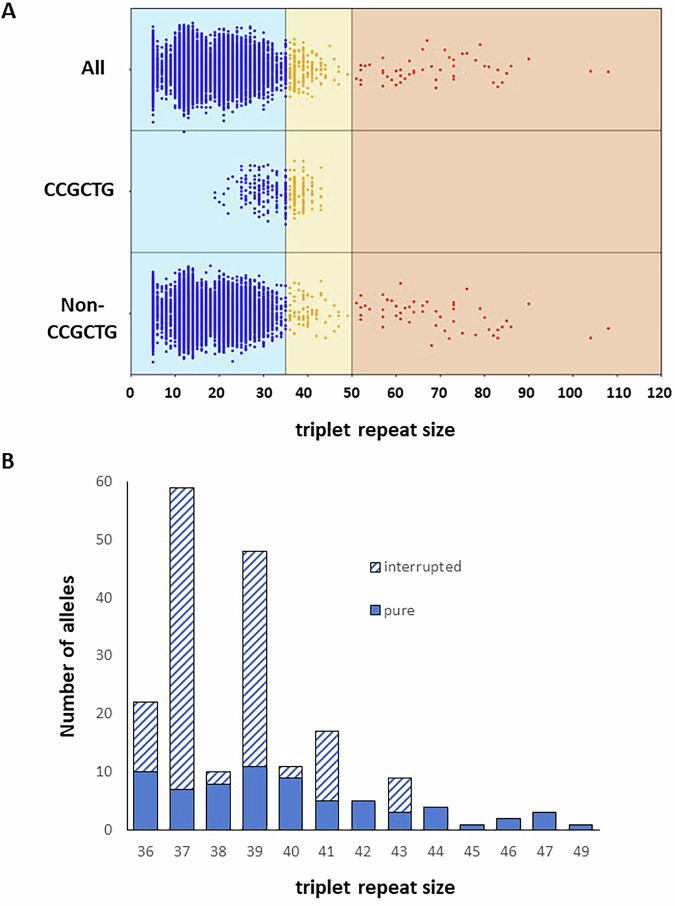
DMPK repeat lengths and CCGCTG interruptions from an exome cohort. **A** Swim lane plot of *DMPK* repeat allele sizes from an exome sequencing cohort that were extracted using ExpansionHunter. For each individual, only the longest allele is shown. The total cohort (All) was separated into alleles with (CCGCTG) and without (bottom lane) CCGCTG interruptions. The repeats are grouped based on non-pathogenic (5–35 repeats, blue dots), intermediate repeats (36–50, yellow dots), and pathogenic ( > 50, red dots). **B** The intermediate alleles from the same cohort are shown by the number of alleles per triplet repeat size. The patterned bars indicate alleles having an interrupted repeat while the solid bars indicate pure CTG repeat. Familial duplicates (from trio-based sequencing) were removed.

The distribution of all alleles in this cohort was as expected, with the majority of alleles having 5, 11, 12, 13, or 14 repeat units (Supplementary Figs. 5). Detection of intermediate and pathogenic alleles in control cohorts has been described before [14–16]. Intermediate (36–50 repeat units) and pathogenic ( > 50 repeat units) alleles showed low percentages, ~0.5% and ~0.1% respectively (Fig. 3A, top). Alleles containing the CCGCTG interruptions are only present in the normal or intermediate range groups (Fig. 3A, middle), with a shift to the lower boundary within the intermediate group (Fig. 3B). More specifically, CCGCTG interrupted alleles were overrepresented in the group of intermediate allele sizes (*n* = 123; 0.3%) and absent from the pathogenic range group (*p* value < 2.2E–16 (CI: 0.0–0.043); Fisher’s Exact test). In contrast, the number of intermediate alleles and pathogenic alleles were similar in the group without the CCGCTG interruption (*n* = 69 versus 52; Fig. 3A, bottom). These data reinforce our observation that CCGCTG interrupted intermediate *DMPK* alleles are not as prone to expansion as pure intermediate alleles.

### CCGCTG interrupted intermediate *DMPK* alleles show a higher intergenerational stability

**Fig. 4:**
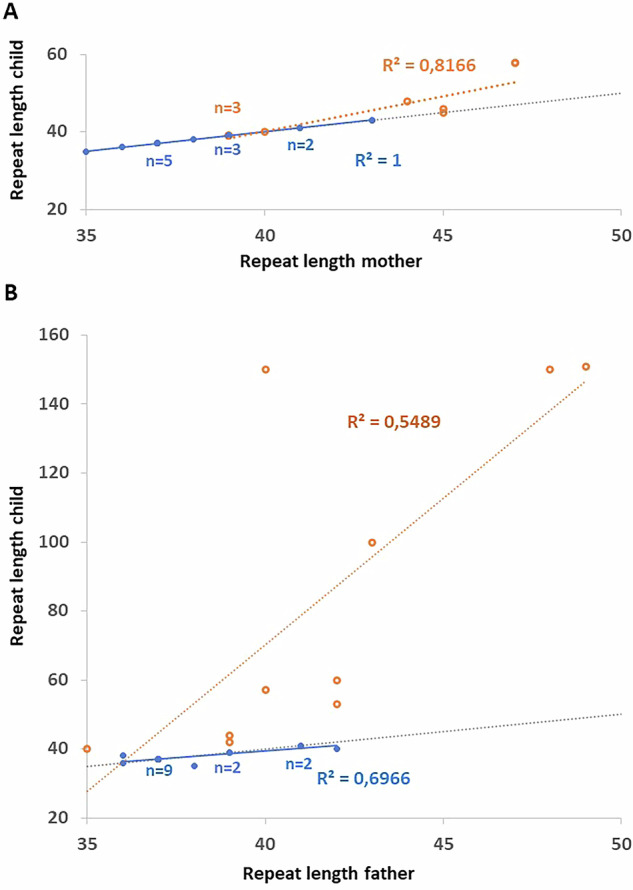
Intergenerational DMPK intermediate repeat length dynamics. The transmissions of intermediate repeats was extracted from the families analysed by targeted testing and exome sequencing data and combined. The intermediate or pathogenic repeat lengths of children were plotted against the intermediate repeat length of their parent. Pure CTG repeats (orang circles) and CCGCTG interrupted repeats (solid blue dots) are shown for maternal (**A**) and paternal (**B**) transmissions. Overlapping data points are indicated by the number of overlaps (n) for pure (above the lines, orange) and interrupted (below the lines, blue) *DMPK* alleles. Lines were created assuming a linear model, R^2^ values are indicated in the graphs. The gray dotted lines indicate hypothetical transmissions with no change in repeat length between parent and child.

The data from the families with intermediate *DMPK* alleles from the exome sequencing dataset was used to analyse transmissions to offspring, stratified by sex. This showed that the CCGCTG interrupted repeat was more stable upon transmission than the pure repeat. As the numbers are small, the data from both cohorts with intermediate repeats (families with a targeted *DMPK* test and the exome sequencing data) were combined to increase power (supplementary table). This showed that the pure CTG repeat was more often increased in size upon transmission. This was just significant for the maternal transmission (Fig. 4A; *p* = 0.03; Fisher’s Exact test), though this is probably biased by the larger size of the pure repeats compared to the interrupted repeats. In contrast, the difference in paternal repeat expansion between pure an interrupted repeats was highly significant (Fig. 4B; *p* = 1.3E–6; Fisher’s Exact test). Paternal expansion bias for intermediate *DMPK* alleles has been observed before [17].

## Discussion

In this study, we showed that CCGCTG interrupted intermediate alleles, total size of 37–43 units, have been detected in multiple families with a history of myotonic dystrophy. However, segregation and microsatellite marker analysis revealed that the individuals with interrupted intermediate alleles have a different haplotype of the *DMPK* locus than affected family members with pathogenic repeat expansions ( > 50 repeat units). In contrast to the pure intermediate alleles, the ones with CCGCTG interruption did not show intergenerational variability in size. These data are consistent with the much more stable CCGCTG interrupted intermediate alleles in semen from a donor [13], and with the three families reported [8, 9, 18]. Furthermore, we showed that the CCGCTG interrupted intermediate alleles have a population frequency of around 0.35% in an exome sequencing data set of >40,000 individuals, representing the general population. In contrast, CCGCTG interruptions were not detected in pathogenic repeat expansions ( > 50 repeats) in our cohort. Again intergenerational instability was only observed in the pure repeats when analyzing trio exome data. Overall, these data show that CCGCTG interrupted *DMPK* alleles with a size of 37–43 units are relatively common and are transmitted to offspring with no increased risk of expansion.

The observation that the *DMPK* intermediate alleles with interruptions are more stable than their pure counterparts, is not unique to myotonic dystrophy. Expansion of the polyglutamine coding CAG repeat in *ATXN1* causes spinocerebellar ataxia type 1. The non-pathogenic CAG repeat is interrupted by one to four CAT (histidine) units, and can be up to 44 repeat units in size. Loss of the CAT interruptions shortens the non-pathogenic allele size to 38 [19]. Similarly, CGG repeat alleles of the *FMR1* gene in the intermediate/premutation range are much less prone to expansion into the pathogenic range, causing fragile X syndrome, when interrupted by one to four AGG units [20].

In contrast to most repeat expansion disorders, where pathogenic alleles are stretches of (almost) pure repeats only, myotonic dystrophy can also be caused by expanded *DMPK* repeats having larger stretches of interruptions [8, 9]. Many different interruption sequences have been described, such as CAG, CTC, CGG, and CCG and can either be in stretches or alternating with one or more CTGs [21]. Although the origin of the interruptions in the *DMPK* repeat are mostly unknown, a few cases were due to the de novo occurrence of interruptions in already existing pathogenic expansions [12]. This is in agreement with a stabilizing effect of the CCGCTG interruption in the intermediate alleles size 37–43, rather than expansion of such interrupted alleles into the pathogenic range.

The risk of further expansions of *DMPK* alleles in subsequent generations, and the associated increase in age at onset and in disease severity, is an incentive for both postnatal and prenatal diagnostic testing for the *DMPK* repeat length. This is not restricted to patients with myotonic dystrophy, but also available for at risk family members, especially in the reproductive age. Additionally, diagnostic testing is offered not only for the pathogenic repeat of more than 50 units, but also for individuals with intermediate alleles of 36–50 repeat units, due to the increased risk of expansion into the pathogenic range upon transmission. Our observation that intermediate alleles of size 37–43 and with CCGCTG interruptions do not have an increased risk of expansion is, thus, of great relevance for individuals with these alleles. This has major implications for genetic testing and counseling as individuals with this repeat and their family members do no longer need cascade testing for a possible risk of DM1. Additionally, these individuals will no longer receive the wrong message of bearing an allele that poses a risk for their offspring.

## Supplementary information

Supplementary fig 1 - Analysis of the DMPK repeat using fragment length analysis of locus-spanning PCR (top figures of each sample A, B, and C) and TP-PCR (bottom figures of each sample A, B, and

Supplementary fig 2 -Pedigrees of DMPK families with interrupted intermediate alleles

Supplementary fig 3 -Pedigrees of DMPK families with pure intermediate alleles

Supplementary fig 4 -Pedigrees of DMPK families with both interrupted and pure intermediate alleles

Supplementary fig 5 -Distribution of DMPK CTG repeat alleles

supplementary JSON file for CGGCAG detection

Supplementary table—allele sizes

## Data Availability

The datasets generated during and/or analysed during the current study are not publicly available due to privacy regulations but (partial) datasets are available from the corresponding author on reasonable request.
