# Supplementary material for: Interrupted CTG repeats in the 37–43 units size range in the 3ʹUTR of DMPK are common alleles
Source: Eur J Hum Genet. 2025 Jul 8;33(11):1547–53. doi: 10.1038/s41431-025-01907-9 (PMC12583562; doi:10.1038/s41431-025-01907-9)
Supplement: Supplementary file 1 — Supplementary fig 1 - Analysis of the DMPK repeat using fragment length analysis of locus-spanning PCR (top figures of each sample A, B, and C) and TP-PCR (bottom figures of each sample A, B, and [file 41431_2025_1907_MOESM1_ESM.pdf]

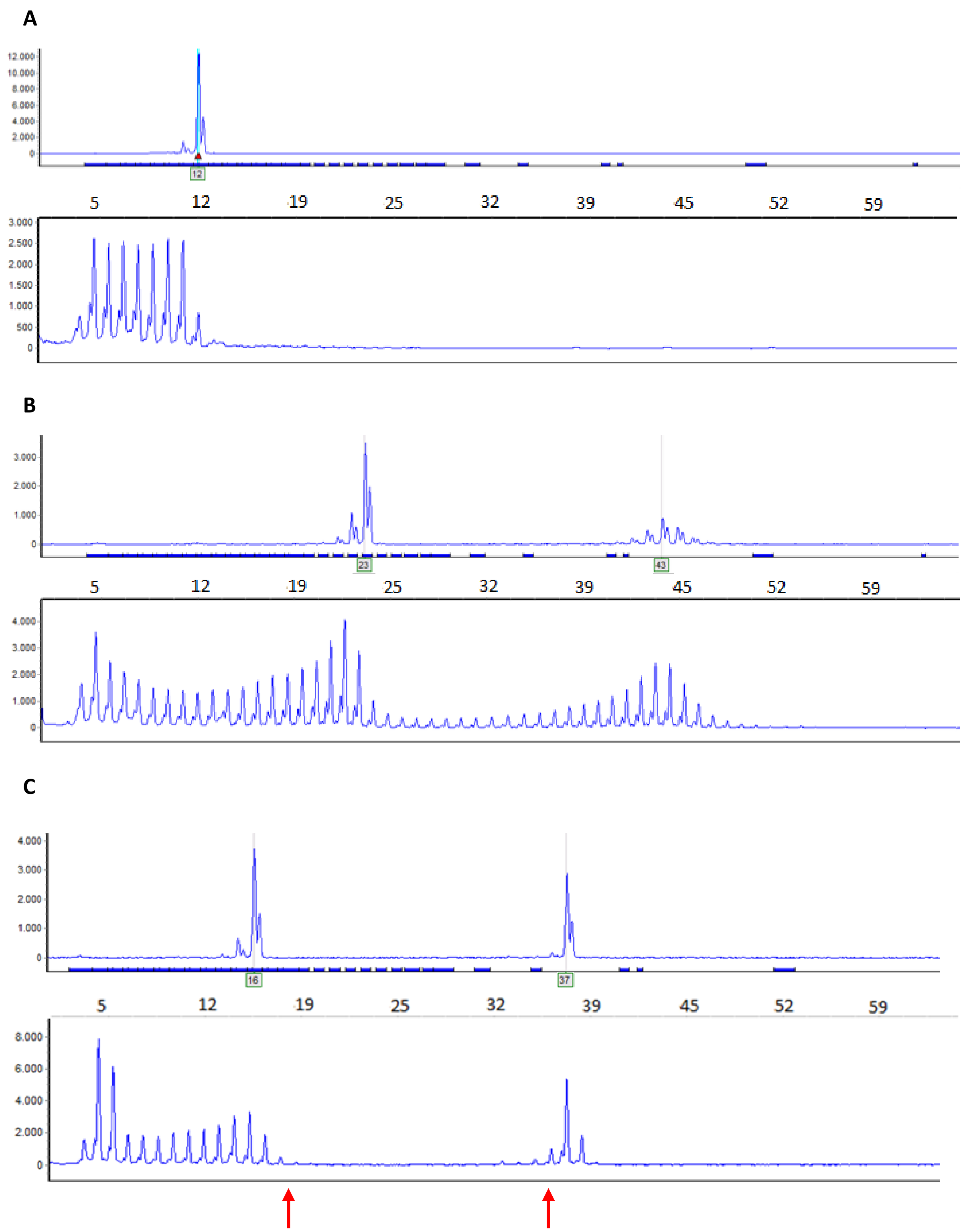

Supplementary Fig. 1: ***Analysis of the DMPK repeat using fragment length analysis of locus-spanning PCR (top figures of each sample A, B, and C) and TP-PCR (bottom figures of each sample A, B, and C).*** *(A) Sample with pure CTG repeats (repeat size of 12, homozygous). (B) sample with two pure CTG repeat (repeat sizes of 23 and 44). (C) Sample with a pure and an interrupted repeat, showing a gap between the red arrows in the middle region of the TP-PCR results (repeat sizes of 16 and 37, respectively).*
