# Supplementary material for: Interrupted CTG repeats in the 37–43 units size range in the 3ʹUTR of DMPK are common alleles
Source: Eur J Hum Genet. 2025 Jul 8;33(11):1547–53. doi: 10.1038/s41431-025-01907-9 (PMC12583562; doi:10.1038/s41431-025-01907-9)
Supplement: Supplementary file 2 — Supplementary fig 2 -Pedigrees of DMPK families with interrupted intermediate alleles [file 41431_2025_1907_MOESM2_ESM.pdf]

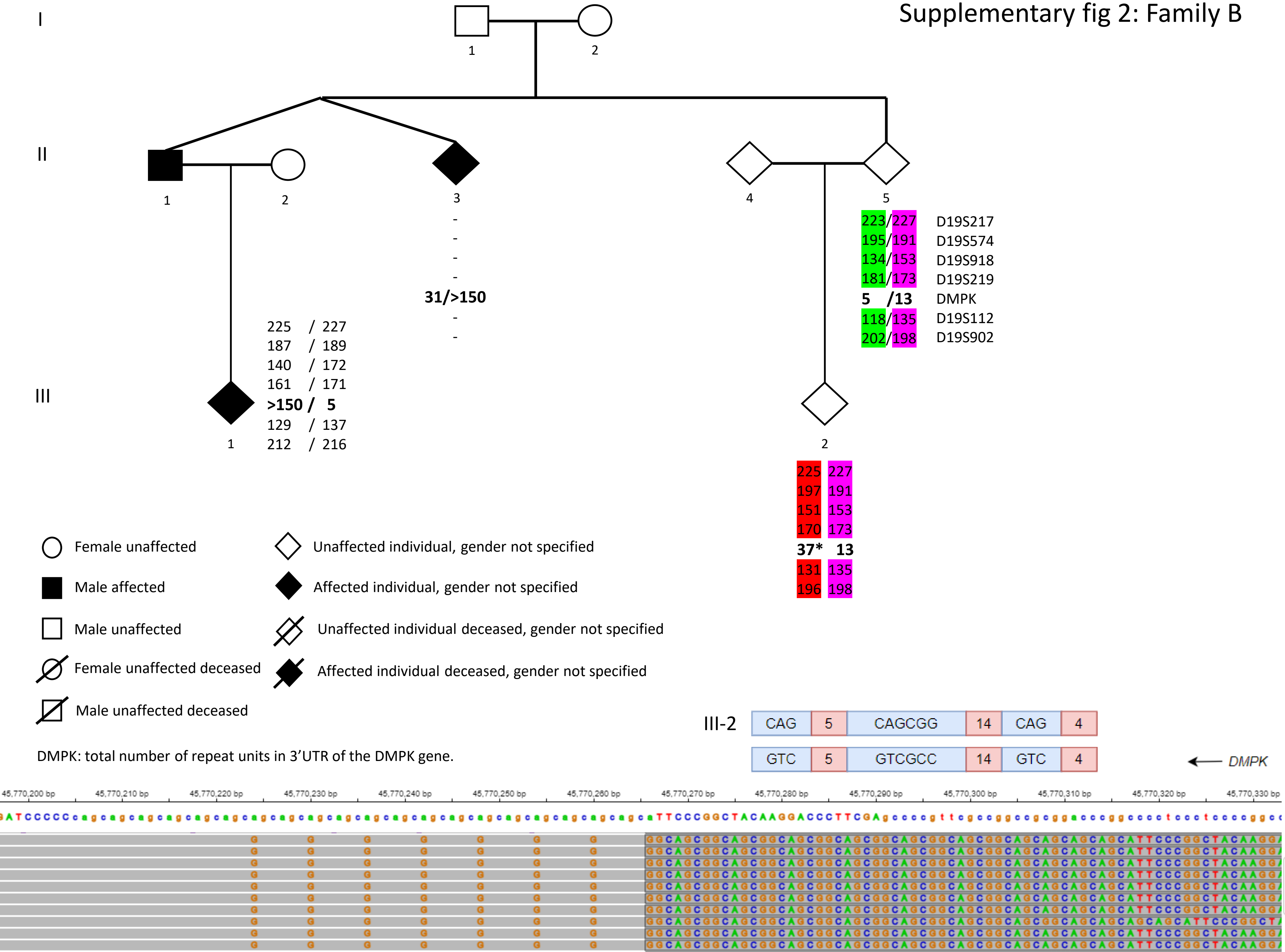

Supplementary fig 2: Family B

Supplementary fig 2: Family B

III-2

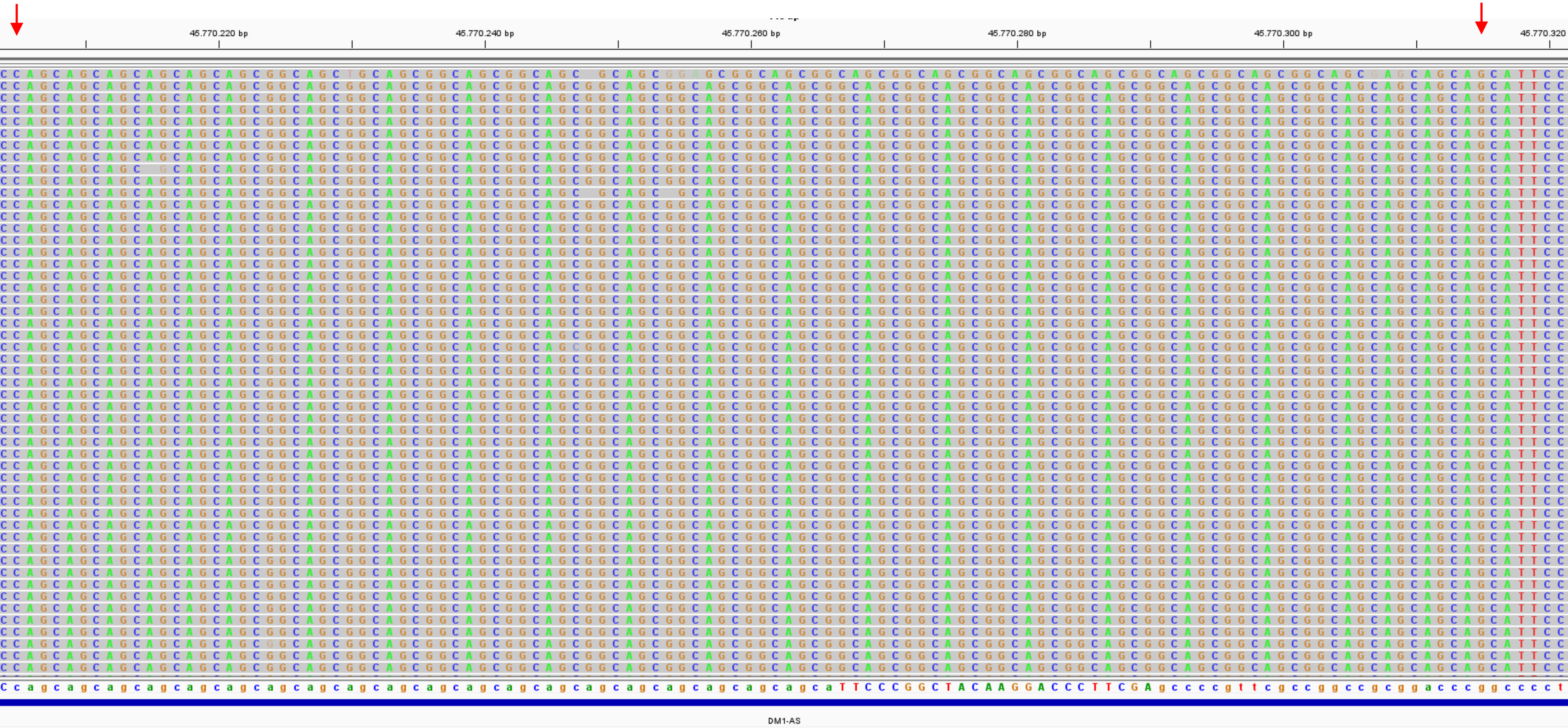

BAM file showing the complete sequence of the DMPK repeat region

Supplementary fig 2: Family C

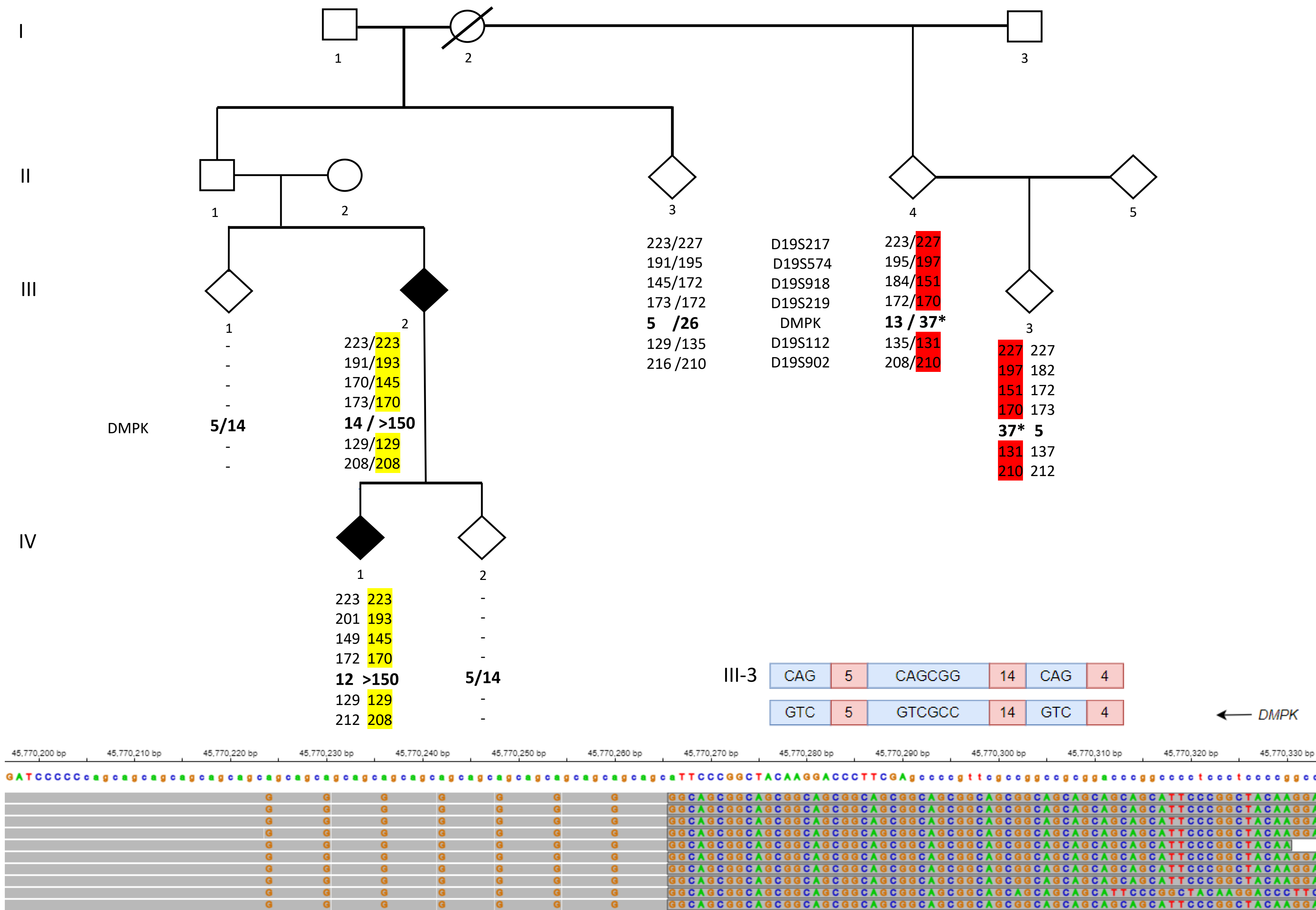

Supplementary fig 2: Family C

III-3

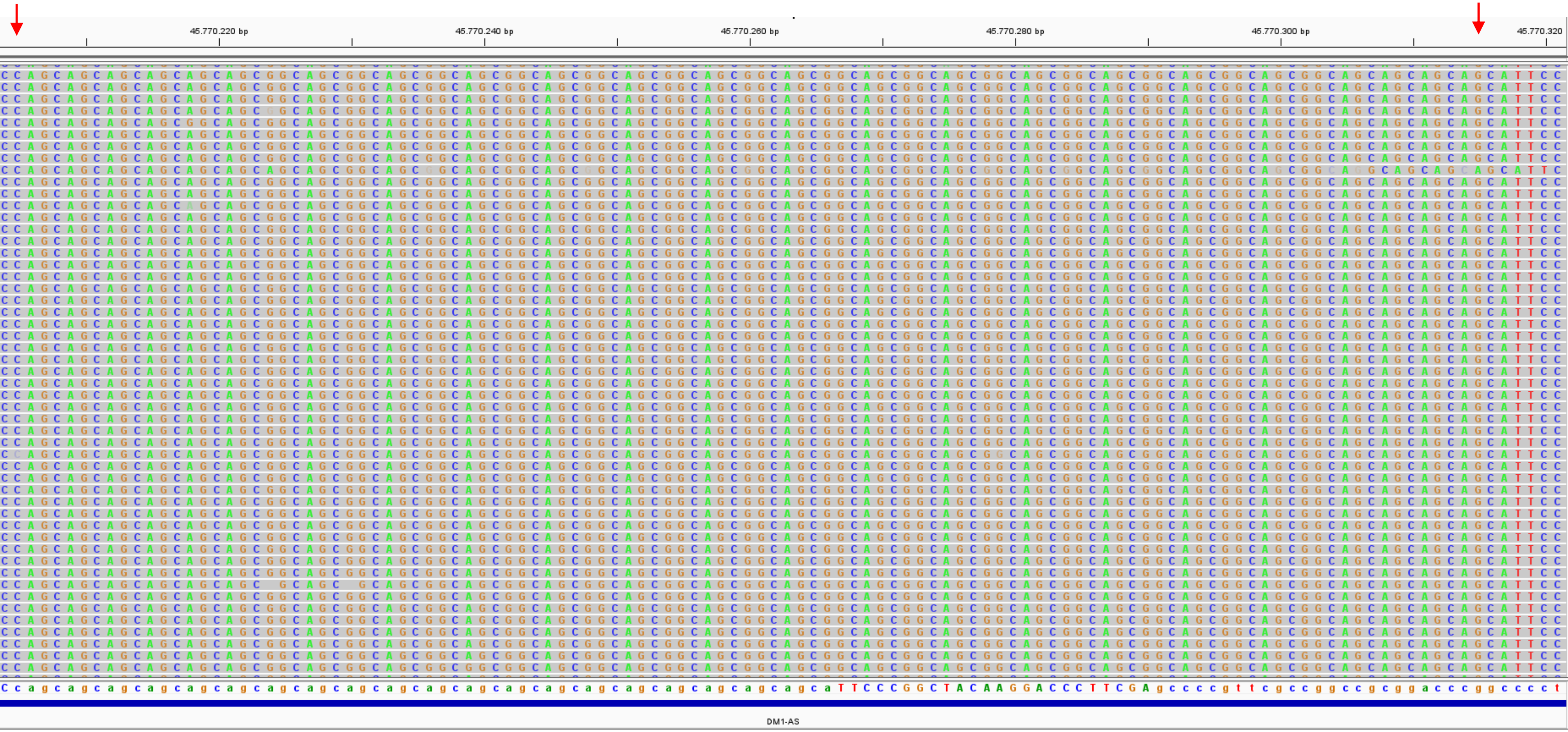

BAM file showing the complete sequence of the DMPK repeat region

Supplementary fig 2: Family D

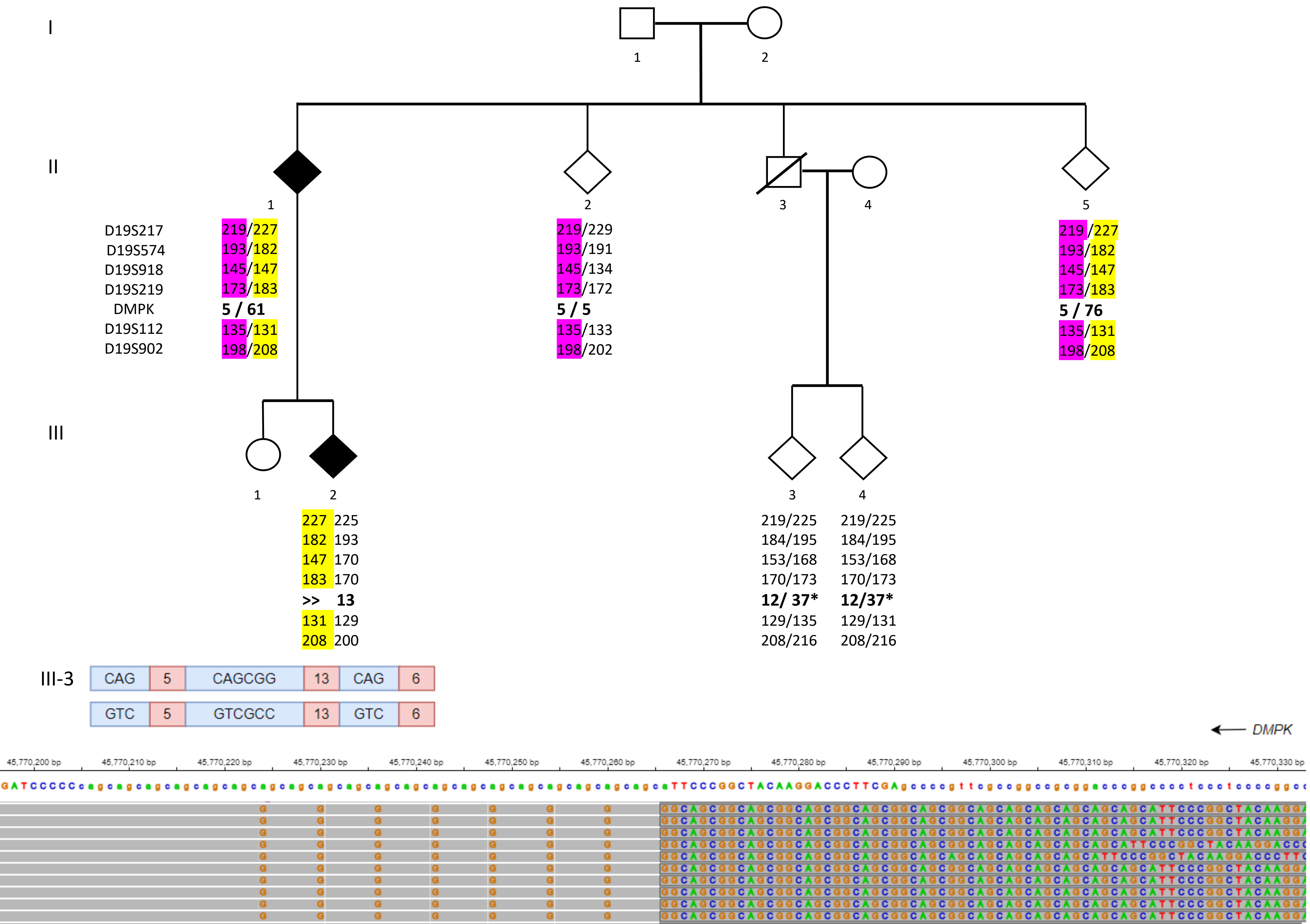

Supplementary fig 2: Family D

III-3

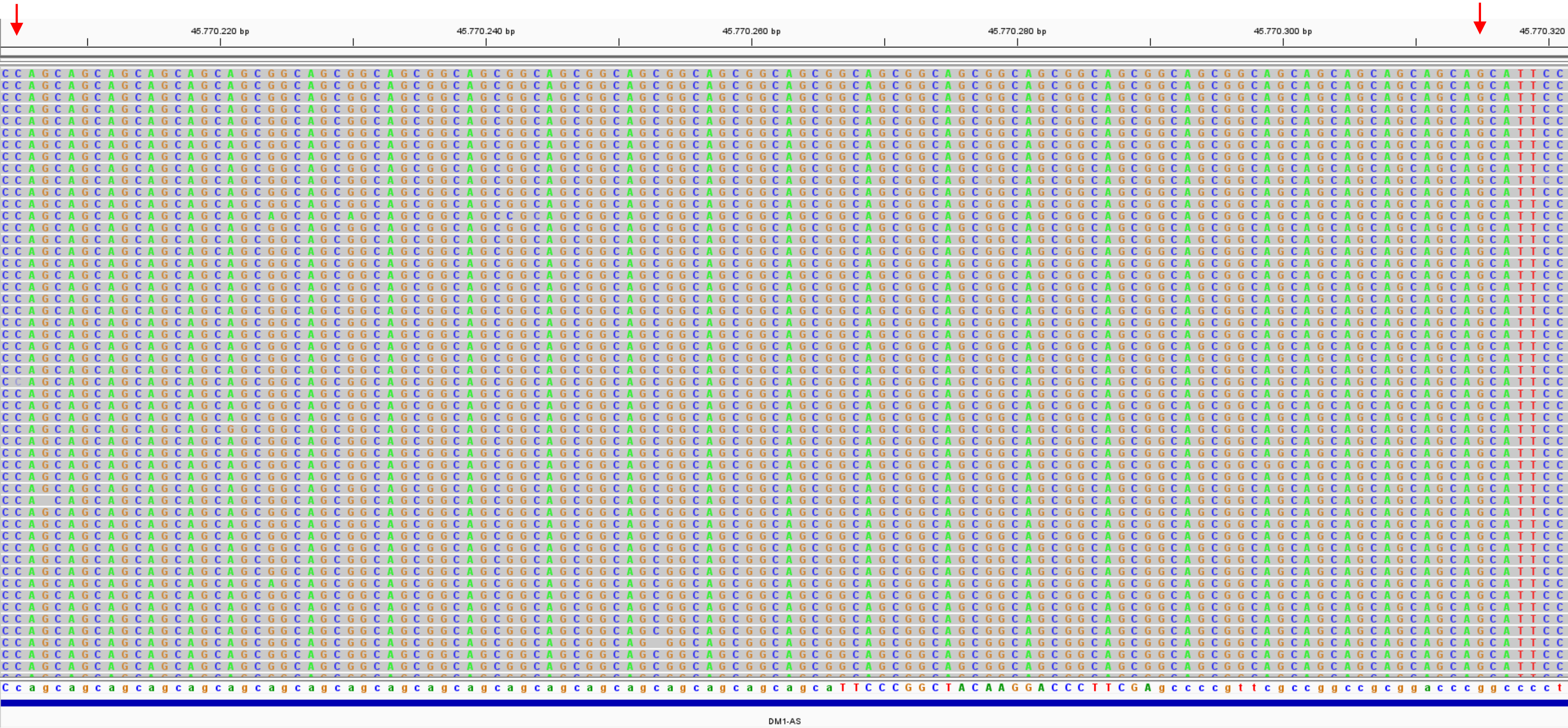

BAM file showing the complete sequence of the DMPK repeat region

Supplementary fig 2: Family E

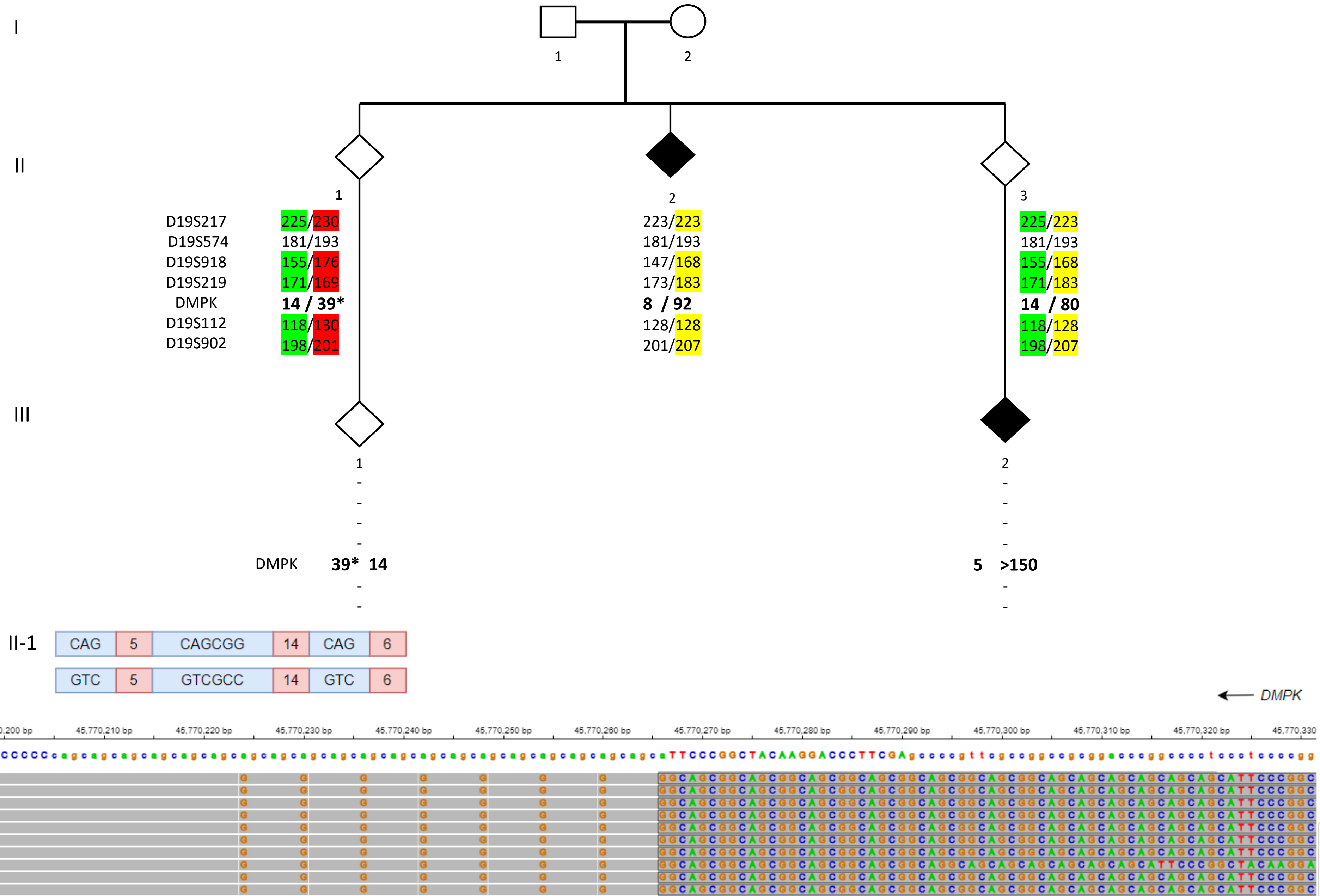

Supplementary fig 2: Family E

II-1

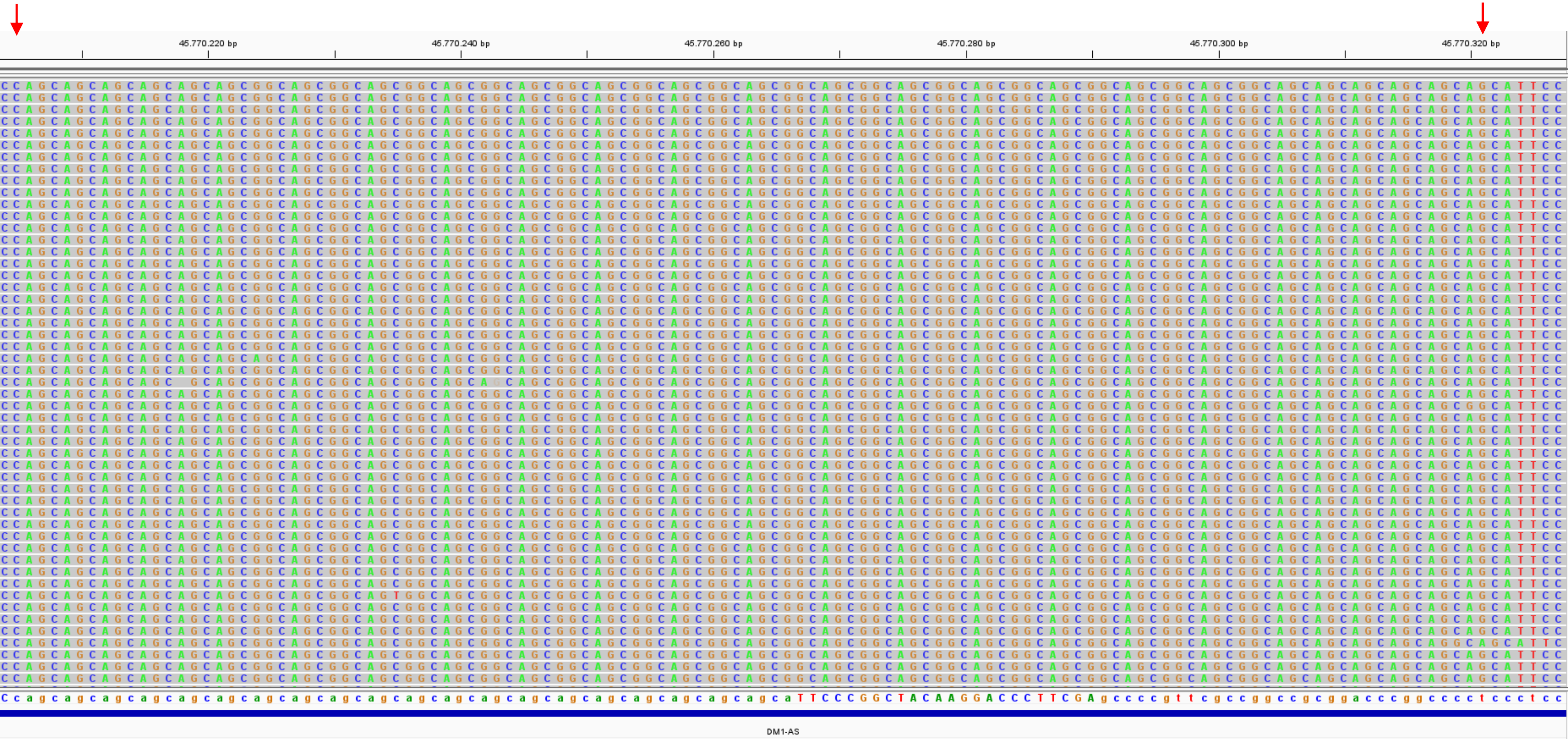

BAM file showing the complete sequence of the DMPK repeat region
