# Supplementary material for: Interrupted CTG repeats in the 37–43 units size range in the 3ʹUTR of DMPK are common alleles
Source: Eur J Hum Genet. 2025 Jul 8;33(11):1547–53. doi: 10.1038/s41431-025-01907-9 (PMC12583562; doi:10.1038/s41431-025-01907-9)
Supplement: Supplementary file 3 — Supplementary fig 3 -Pedigrees of DMPK families with pure intermediate alleles [file 41431_2025_1907_MOESM3_ESM.pdf]

Supplementary fig 3: Family G

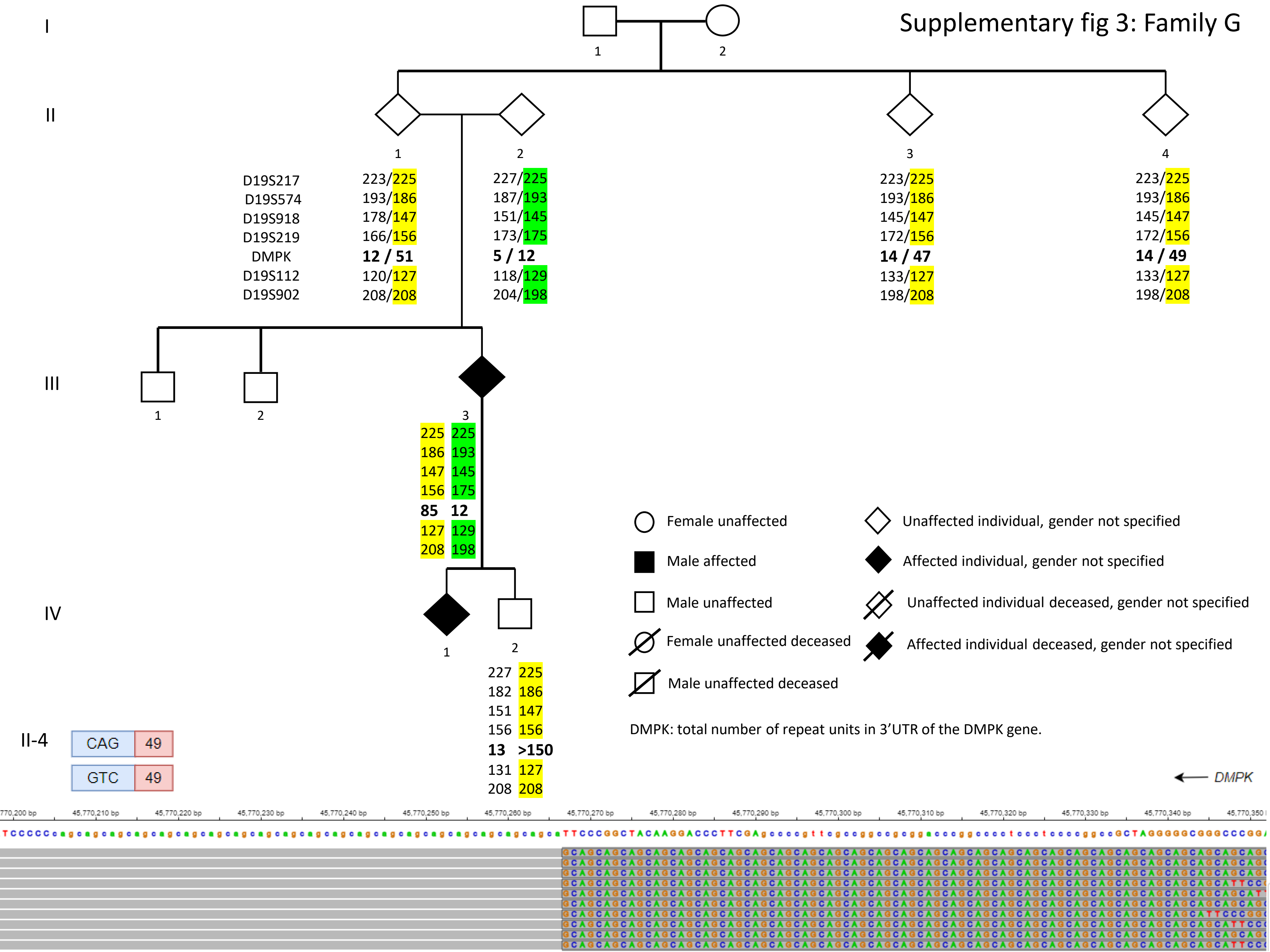

Supplementary fig 3: Family G

II-4

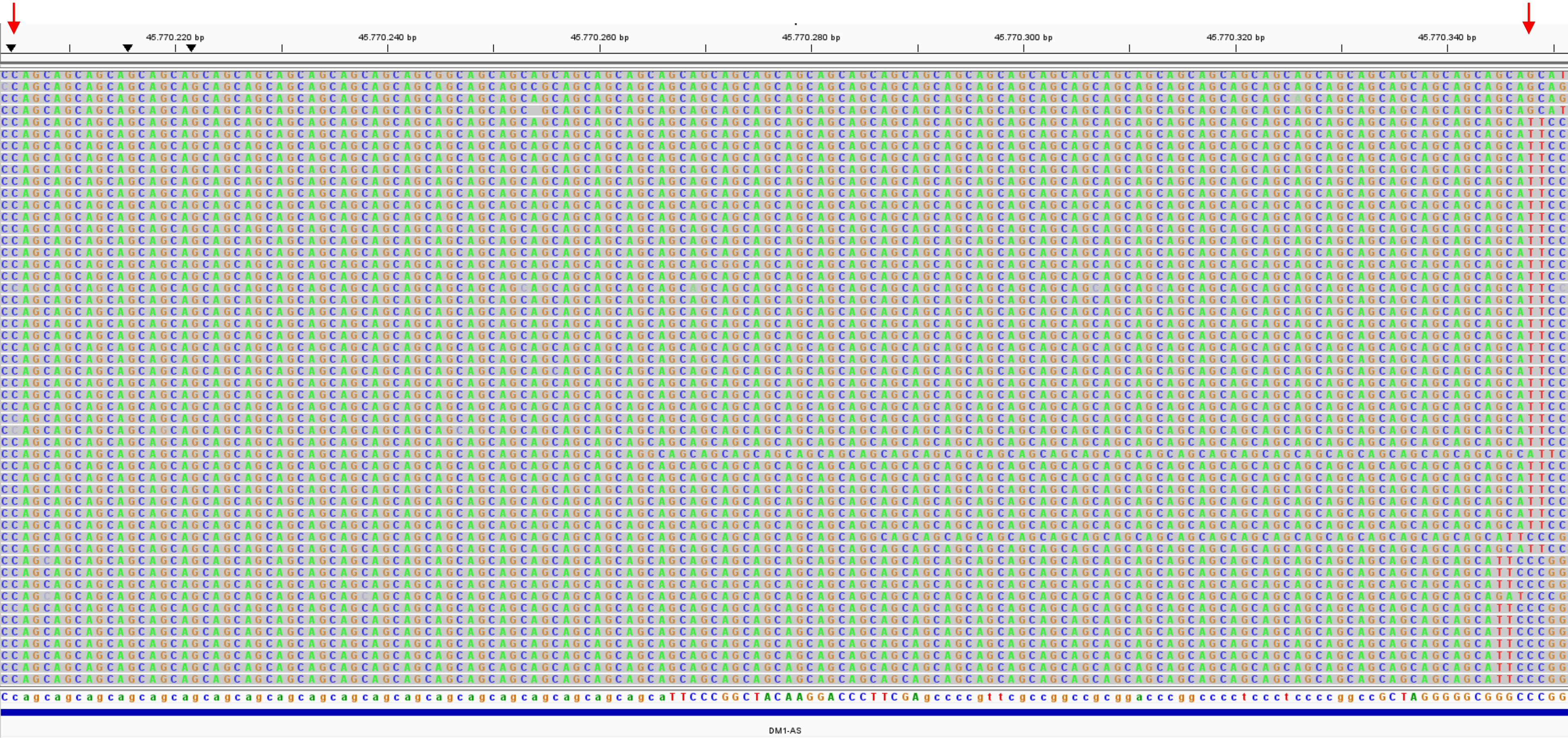

BAM file showing the complete sequence of the DMPK repeat region

Supplementary fig 3: Family H

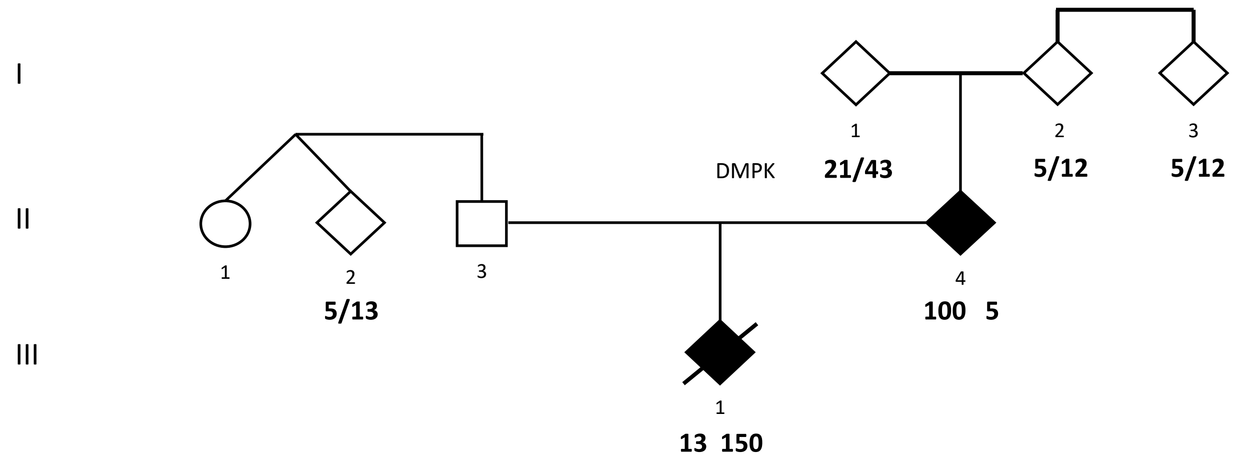

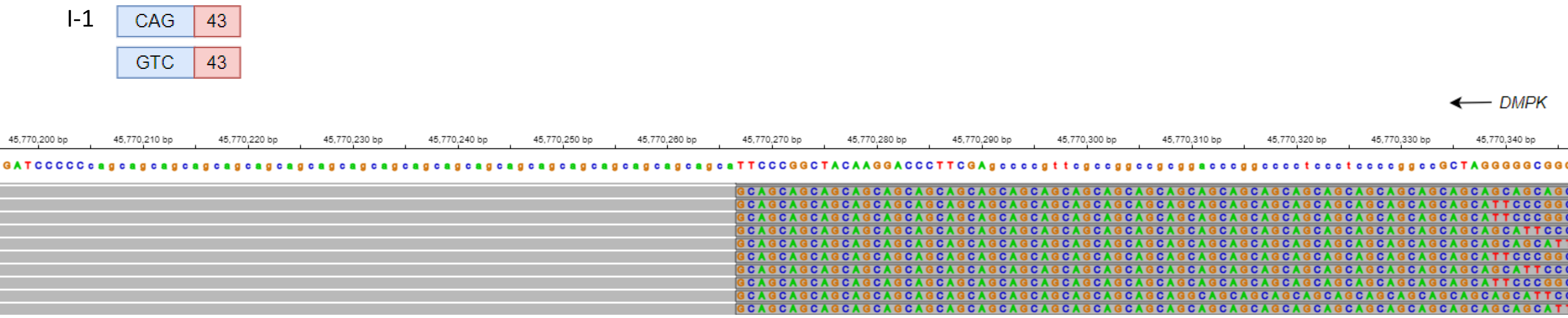

Supplementary fig 3: Family H

I-1

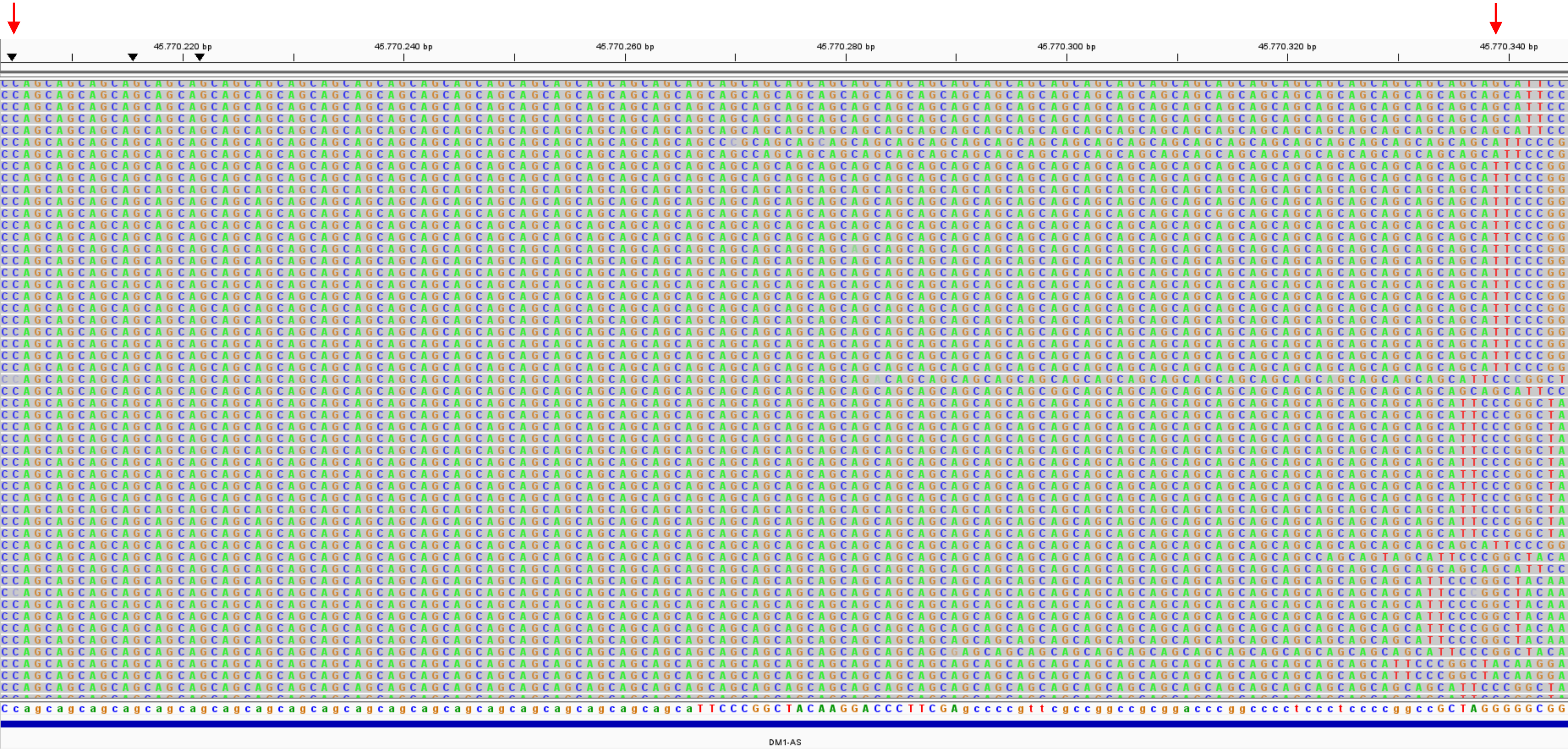

BAM file showing the complete sequence of the DMPK repeat region

Supplementary fig 3: Family I

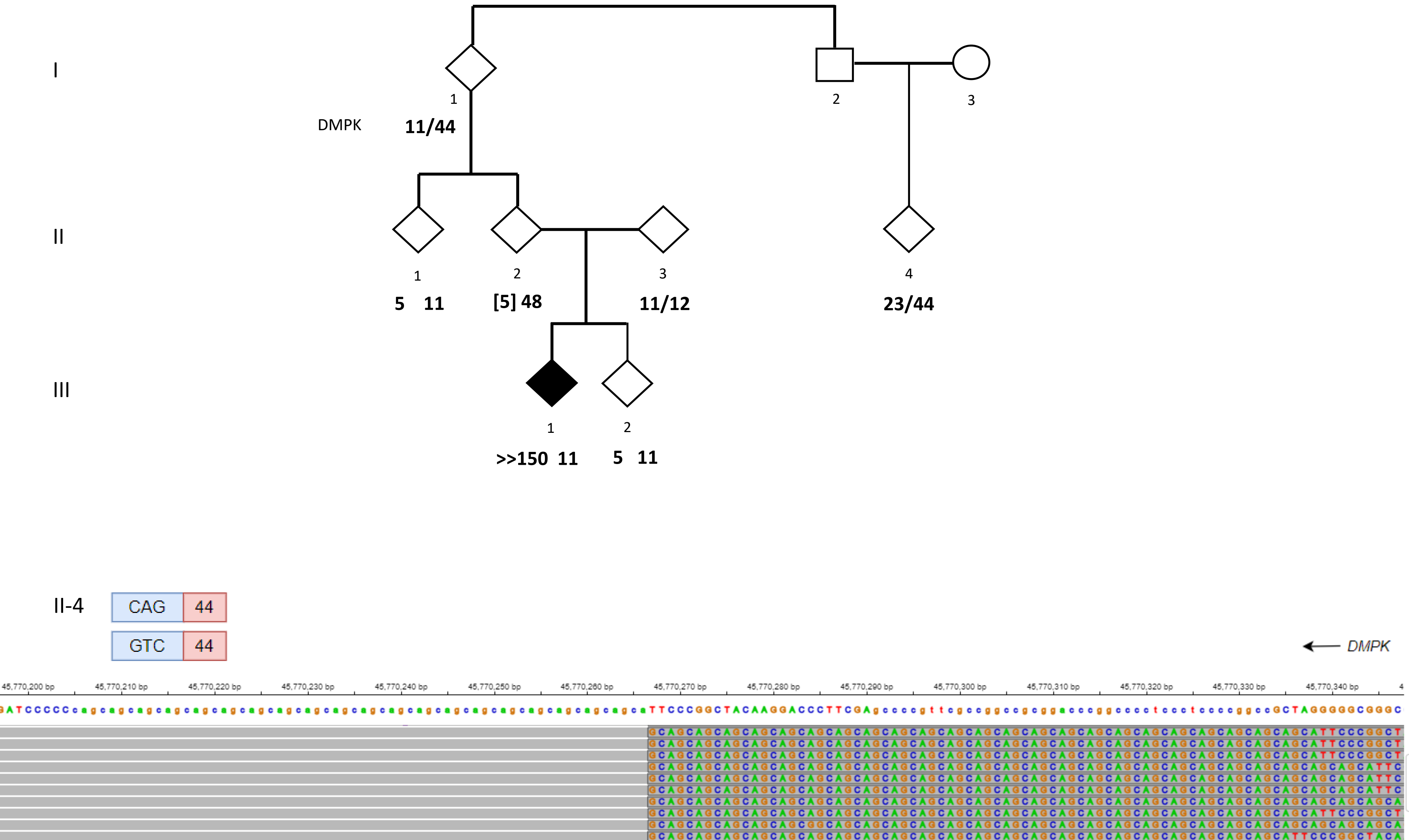

Supplementary fig 3: Family I

II-4

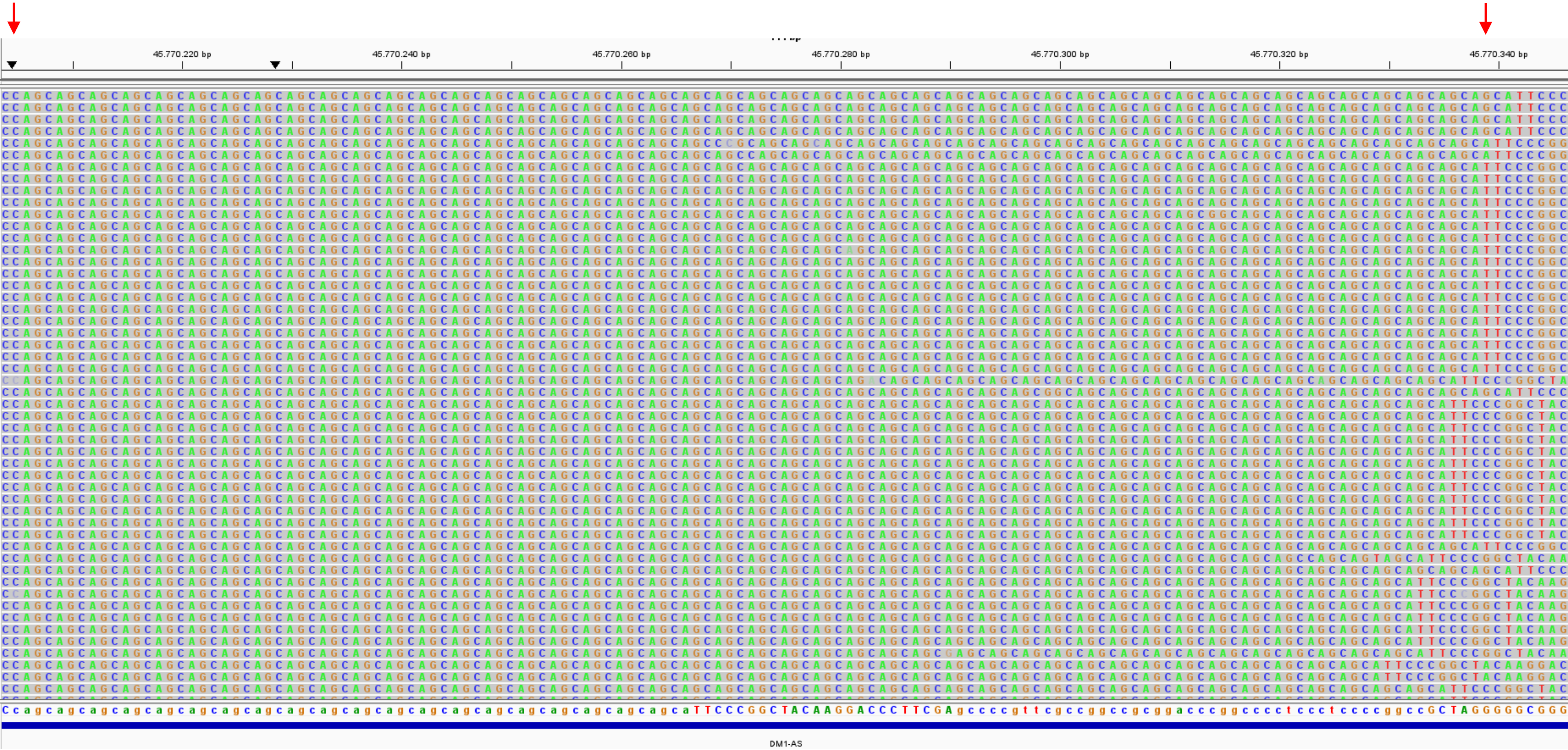

BAM file showing the complete sequence of the DMPK repeat region

Supplementary fig 3: Family J

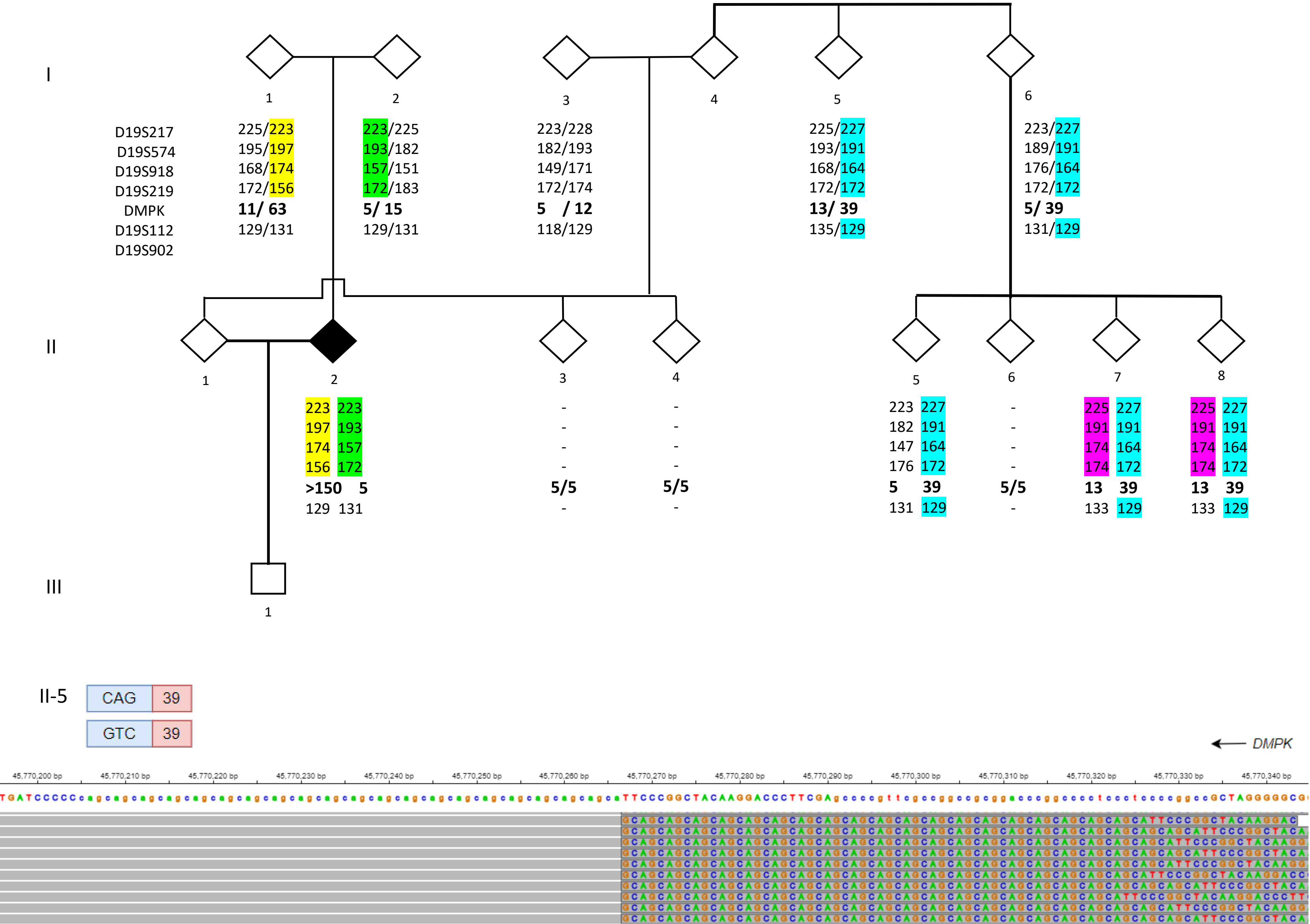

II-5

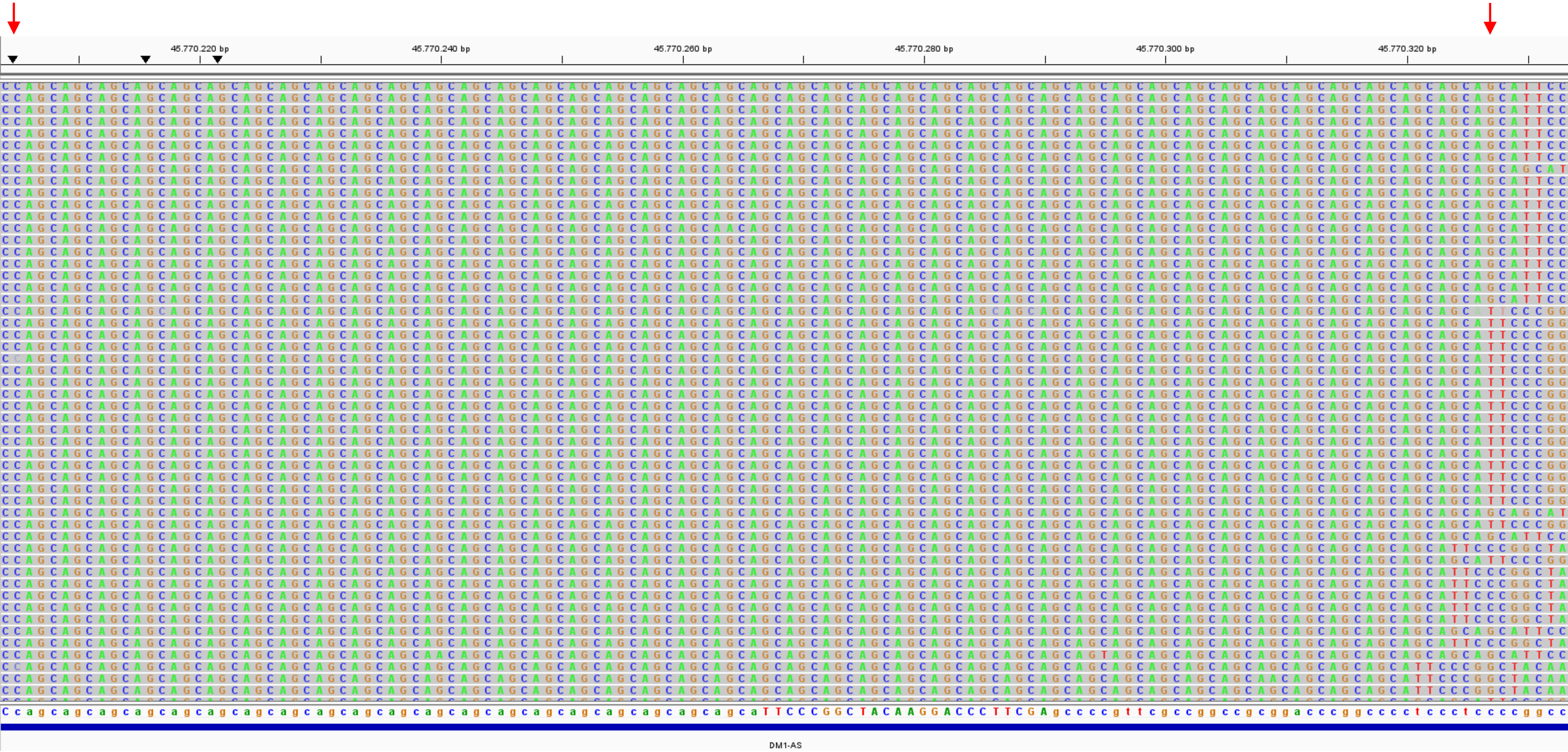

BAM file showing the complete sequence of the DMPK repeat region

Supplementary fig 3: Family K

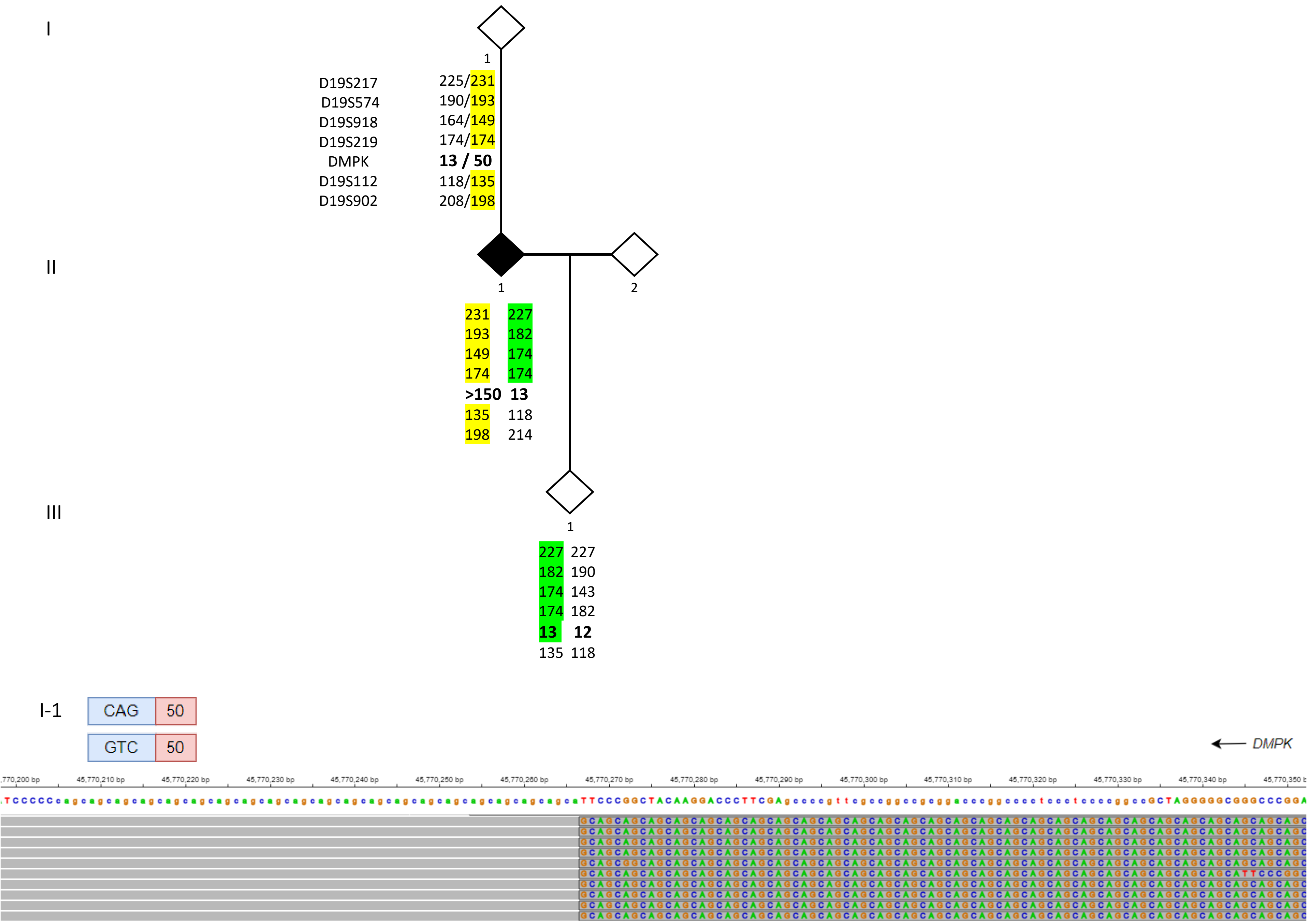

Supplementary fig 3: Family K

I-1

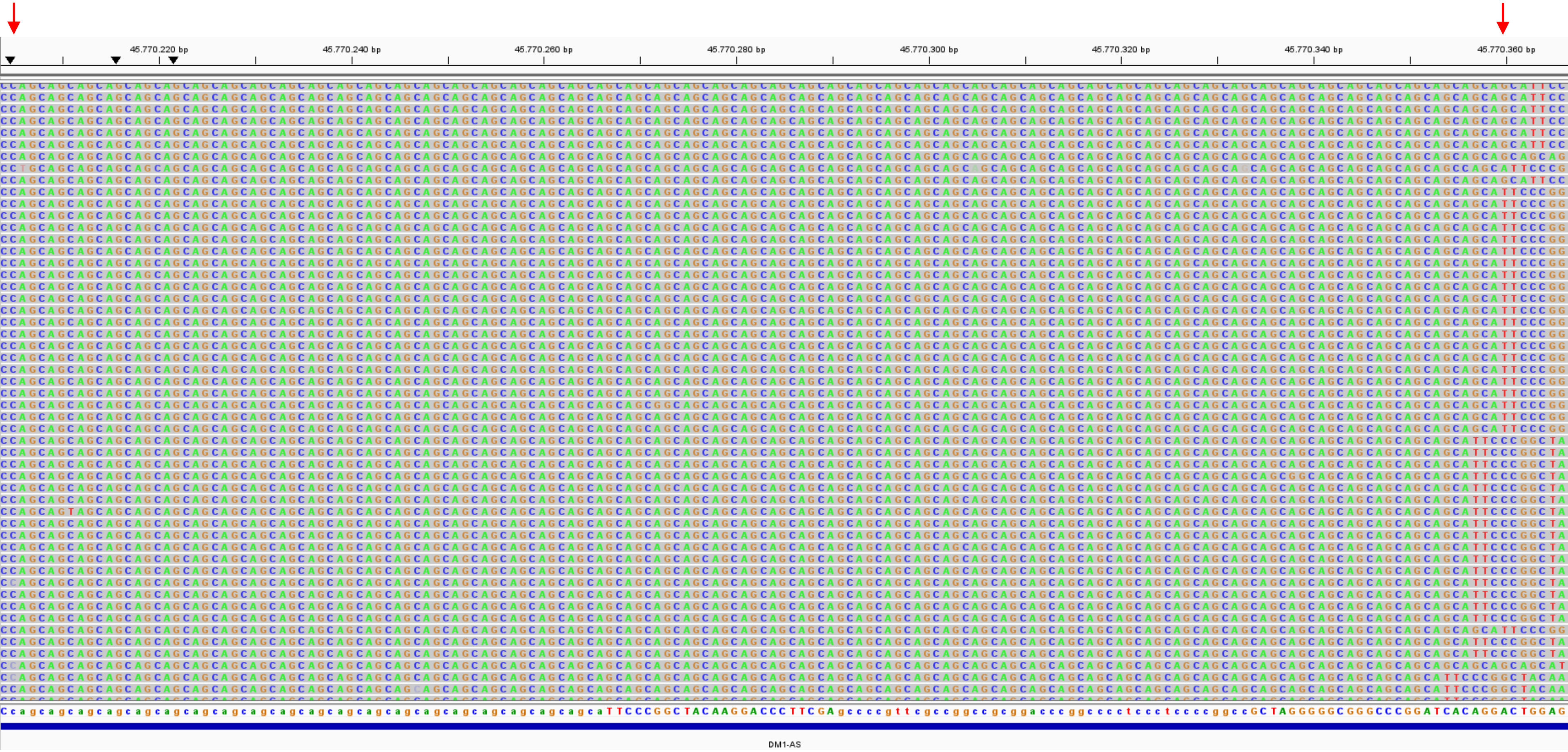

BAM file showing the complete sequence of the DMPK repeat region

Supplementary fig 3: Family L

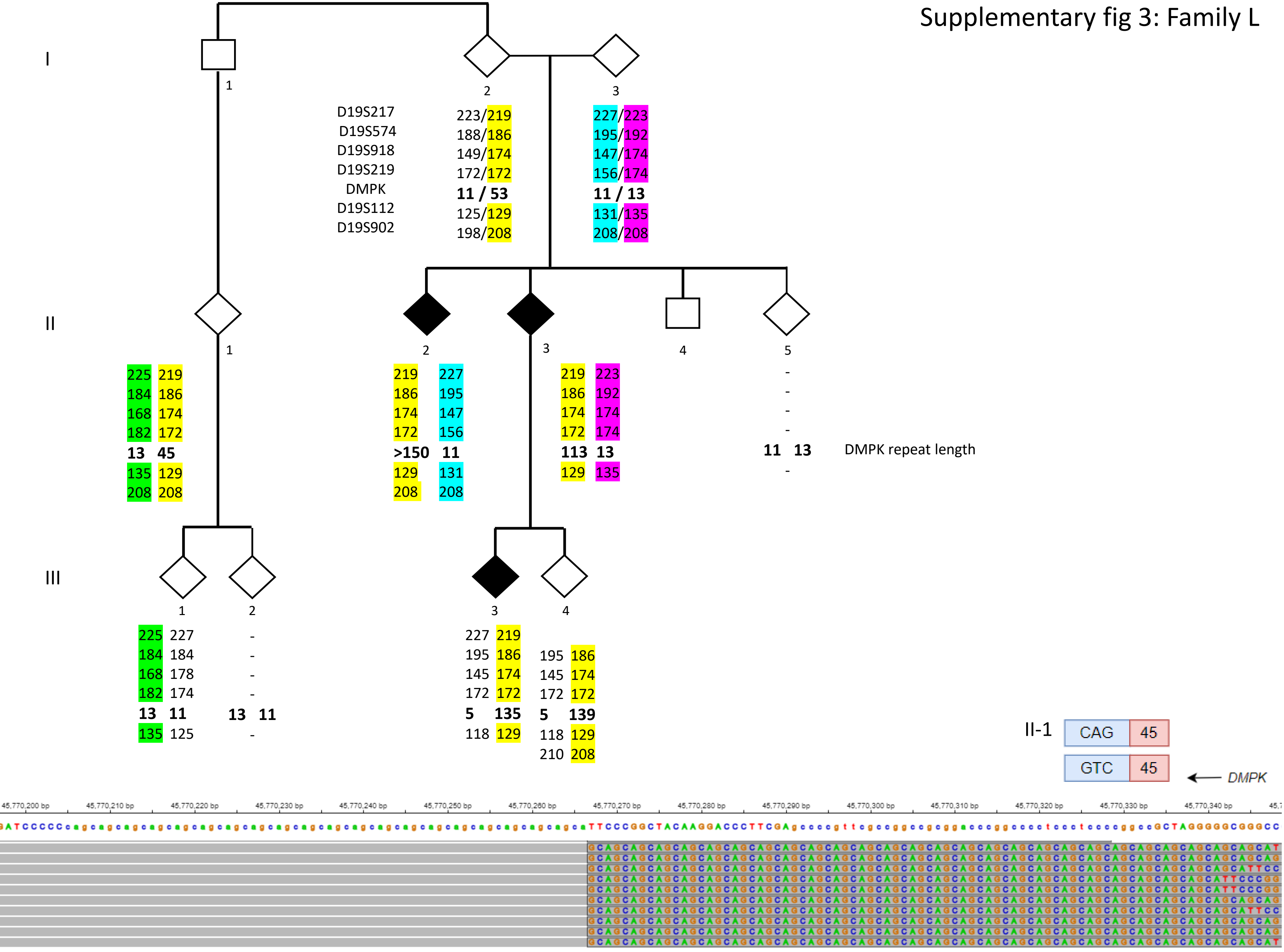

Supplementary fig 3: Family L

II-1

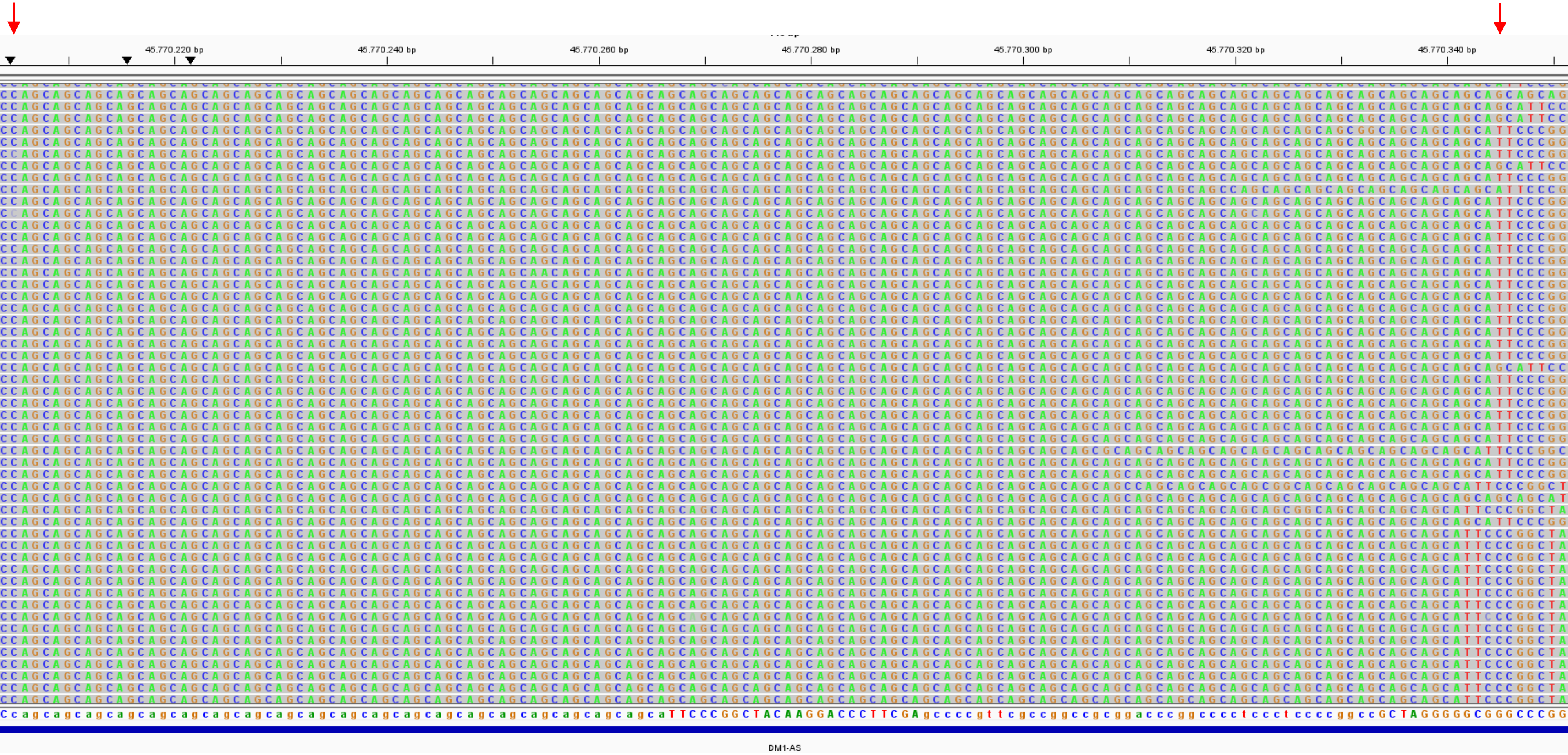

BAM file showing the complete sequence of the DMPK repeat region

Supplementary fig 3: Family M

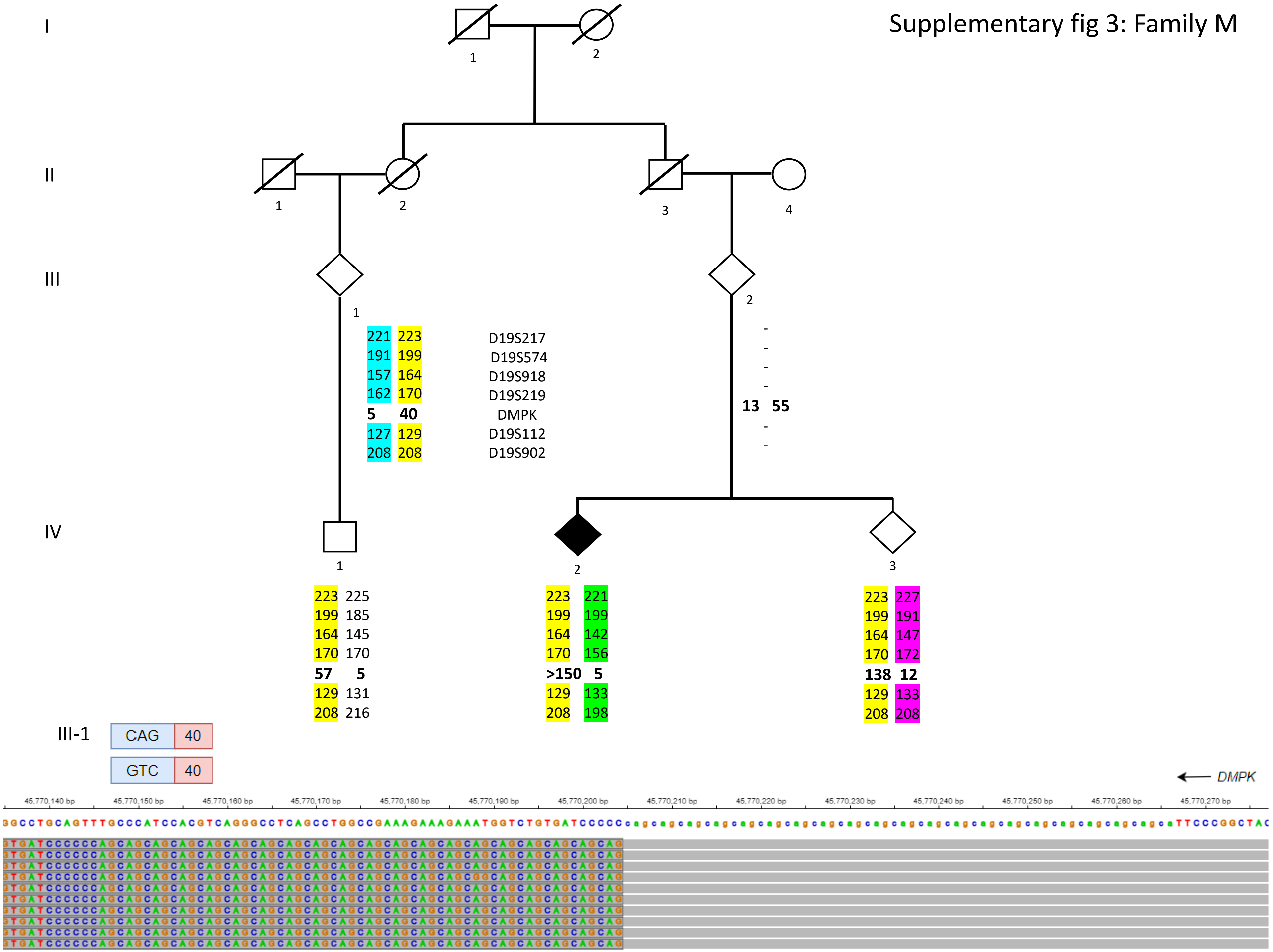

Supplementary fig 3: Family M

III-1

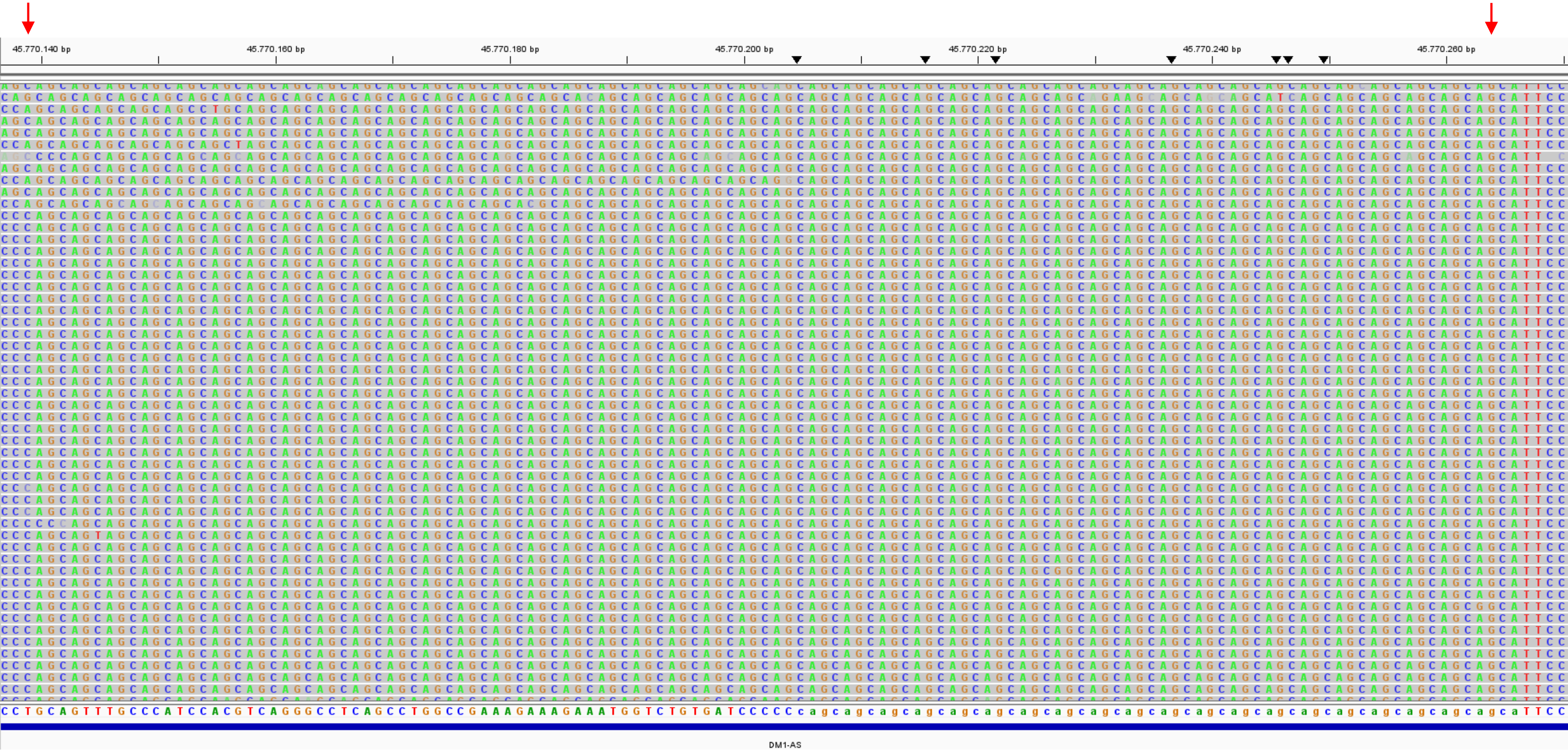

BAM file showing the complete sequence of the DMPK repeat region

Supplementary fig 3: Family N

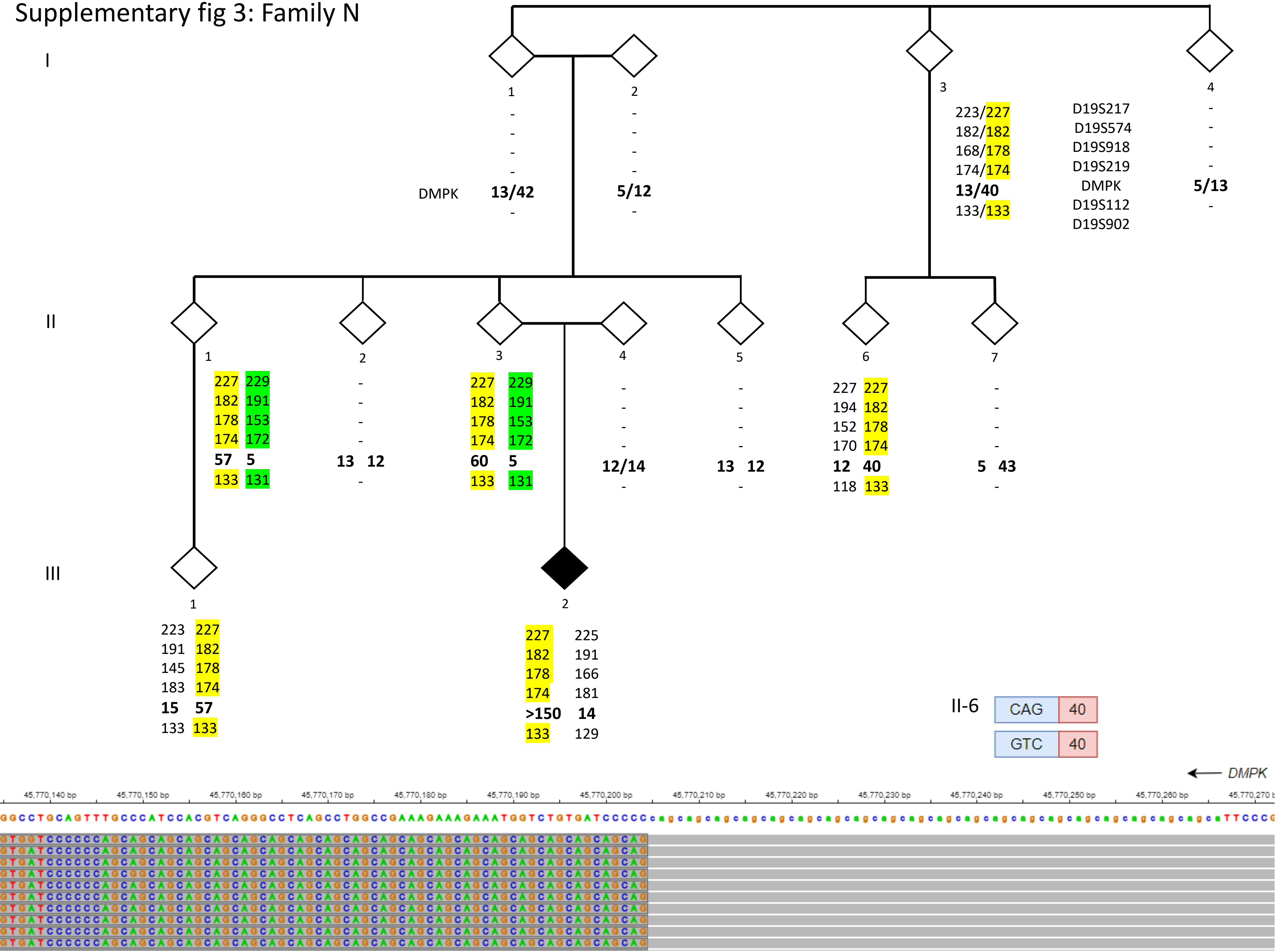

Supplementary fig 3: Family N

II-6

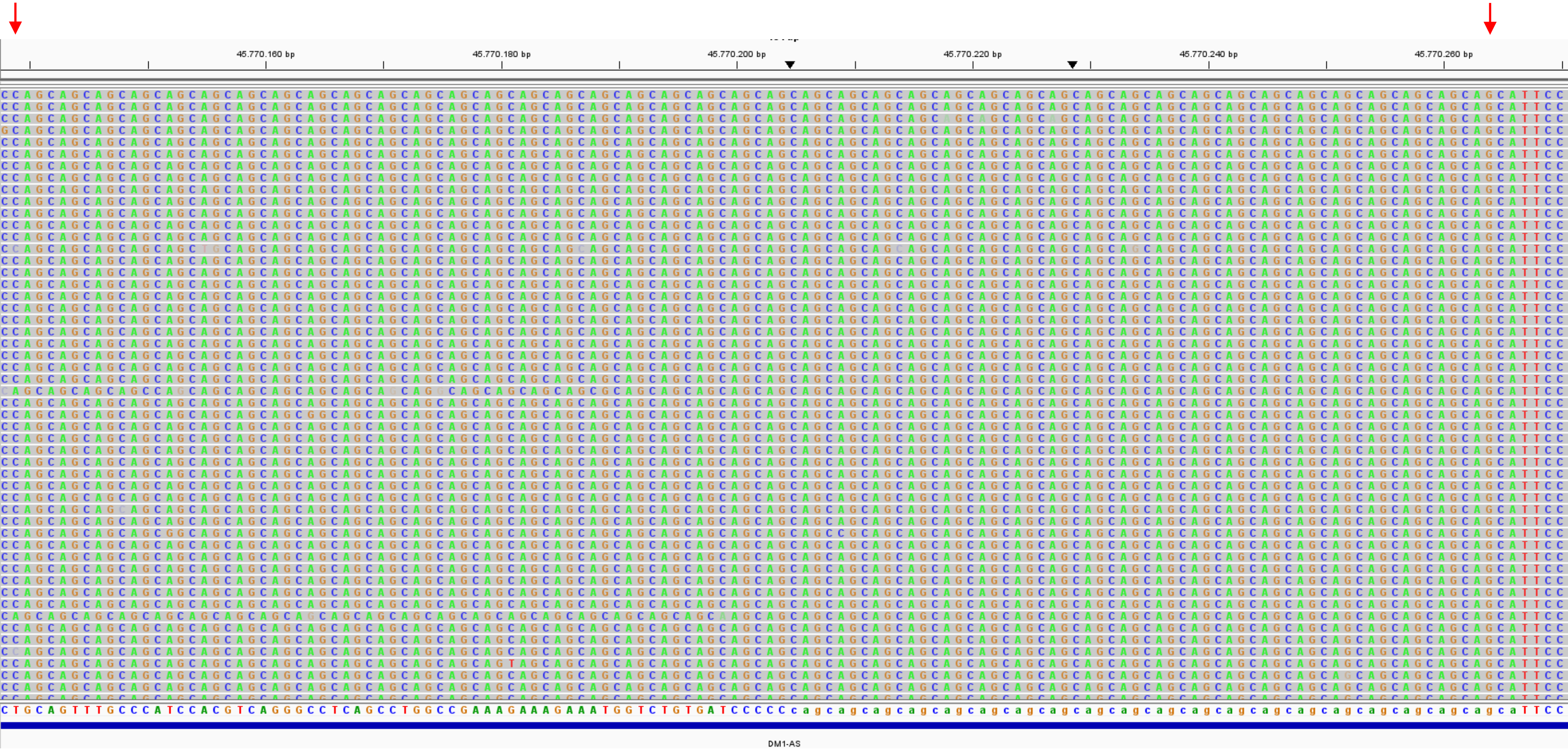

BAM file showing the complete sequence of the DMPK repeat region
