# Supplementary material for: Interrupted CTG repeats in the 37–43 units size range in the 3ʹUTR of DMPK are common alleles
Source: Eur J Hum Genet. 2025 Jul 8;33(11):1547–53. doi: 10.1038/s41431-025-01907-9 (PMC12583562; doi:10.1038/s41431-025-01907-9)
Supplement: Supplementary file 4 — Supplementary fig 4 -Pedigrees of DMPK families with both interrupted and pure intermediate alleles [file 41431_2025_1907_MOESM4_ESM.pdf]

Supplementary fig 4: Family A (same figure as in article)

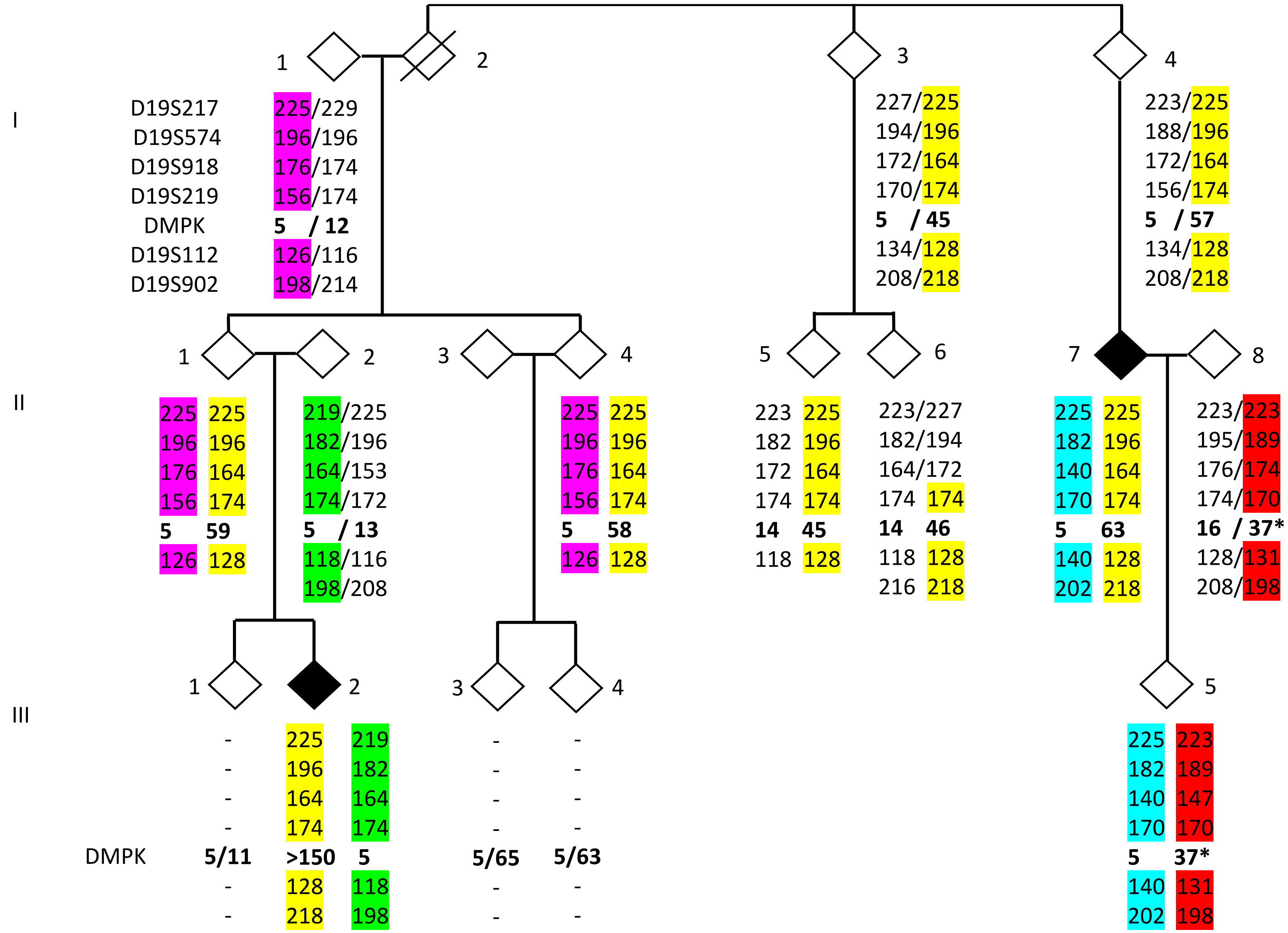

Supplementary fig 4: Family A

- Female unaffected
- Male affected
- Male unaffected
- Female unaffected deceased
- Male unaffected deceased
- Unaffected individual, gender not specified
- Affected individual, gender not specified
- Unaffected individual deceased, gender not specified
- Affected individual deceased, gender not specified

DMPK: total number of repeat units in 3'UTR of the DMPK gene.

Supplementary fig 4: Family A (same figure as in article)

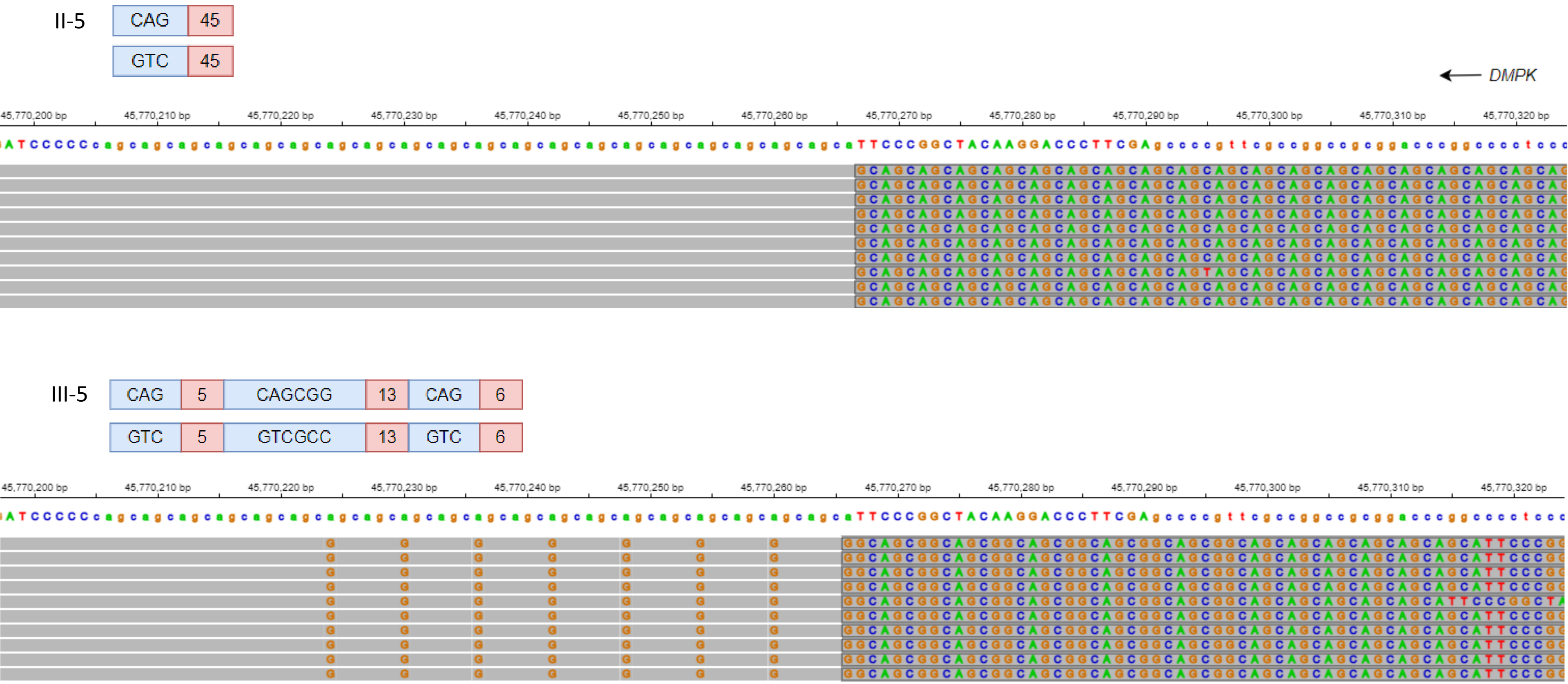

Supplementary fig 4: Family A

II-5

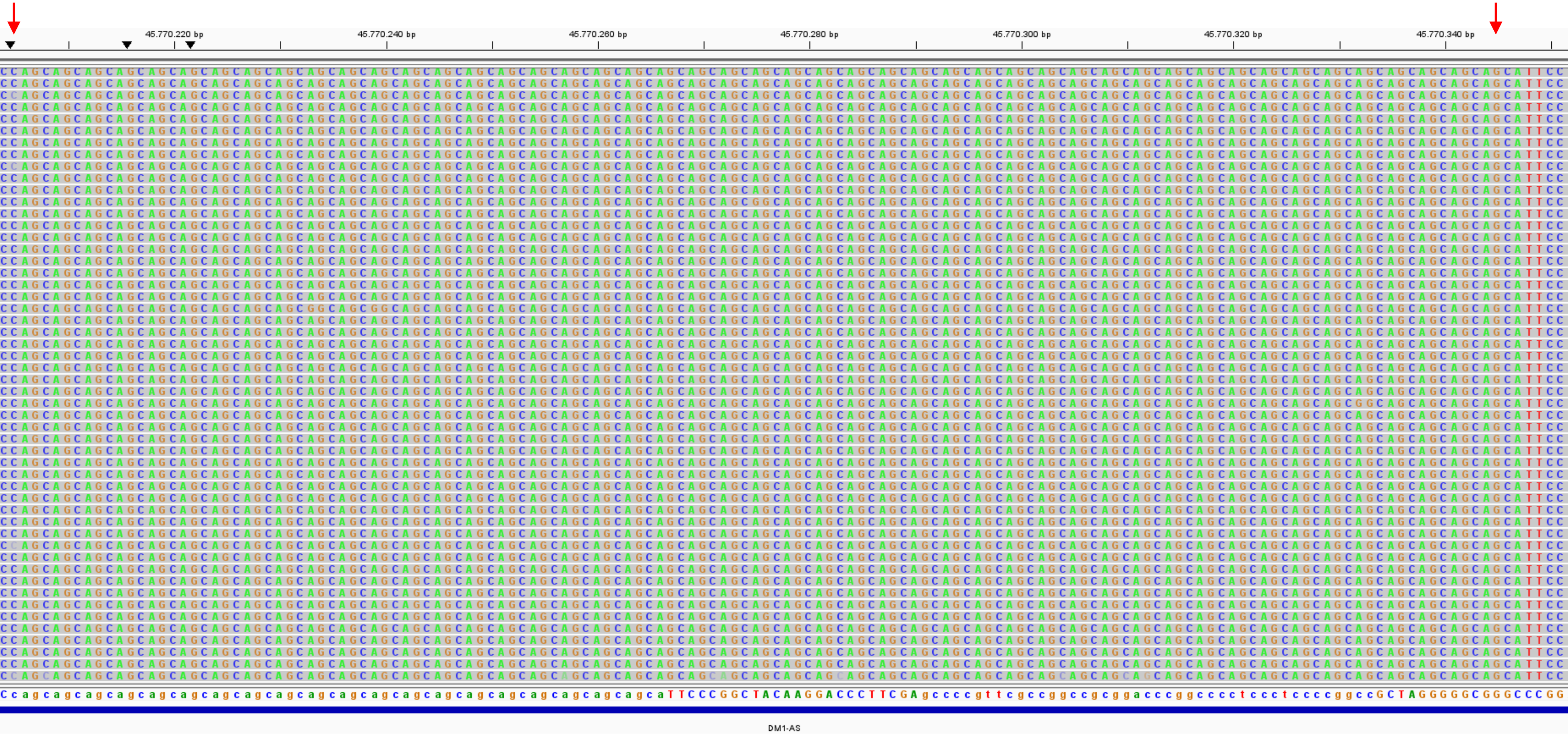

BAM file showing the complete sequence of the DMPK repeat region

Supplementary fig 4: Family A

III-5

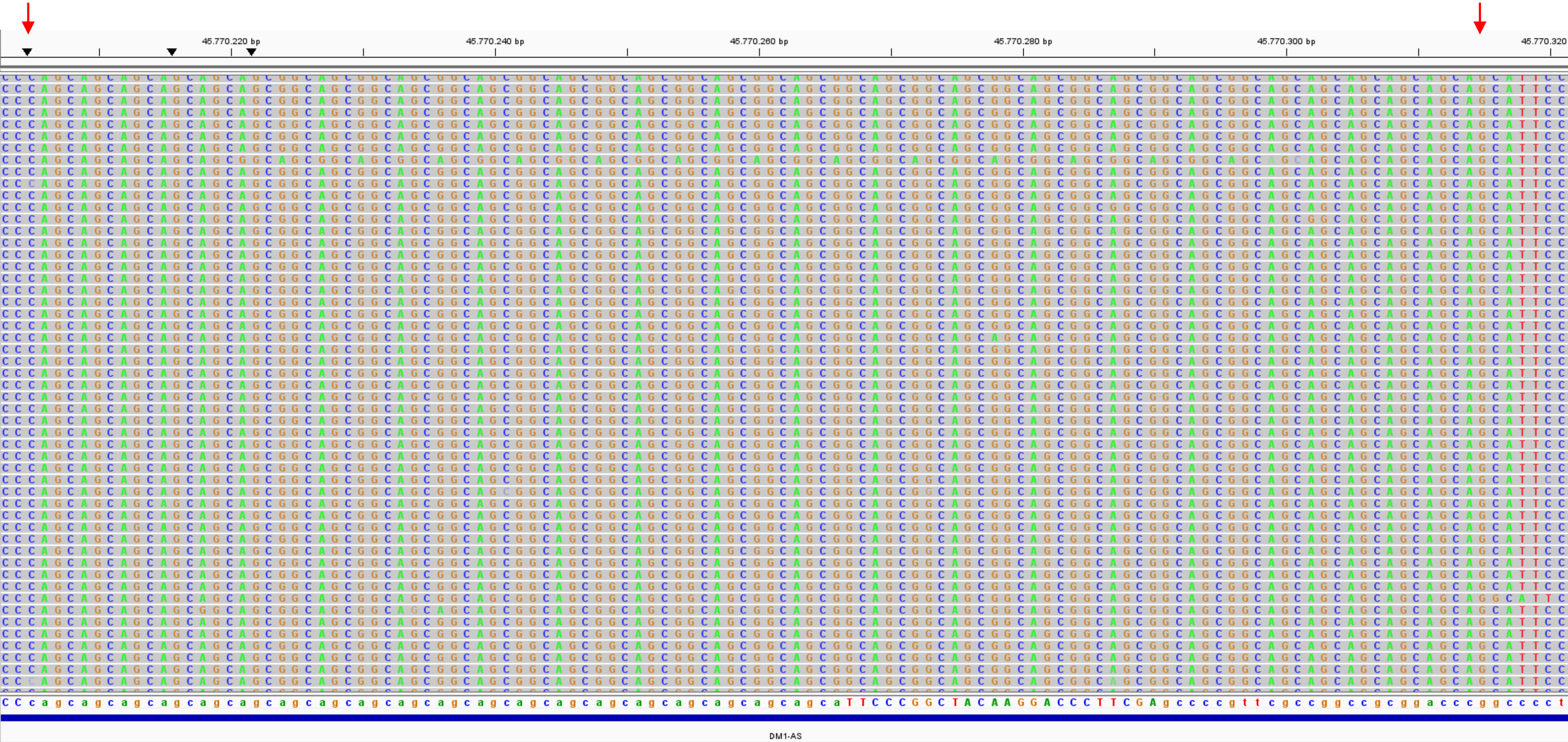

BAM file showing the complete sequence of the DMPK repeat region

Supplementary fig 4: Family F

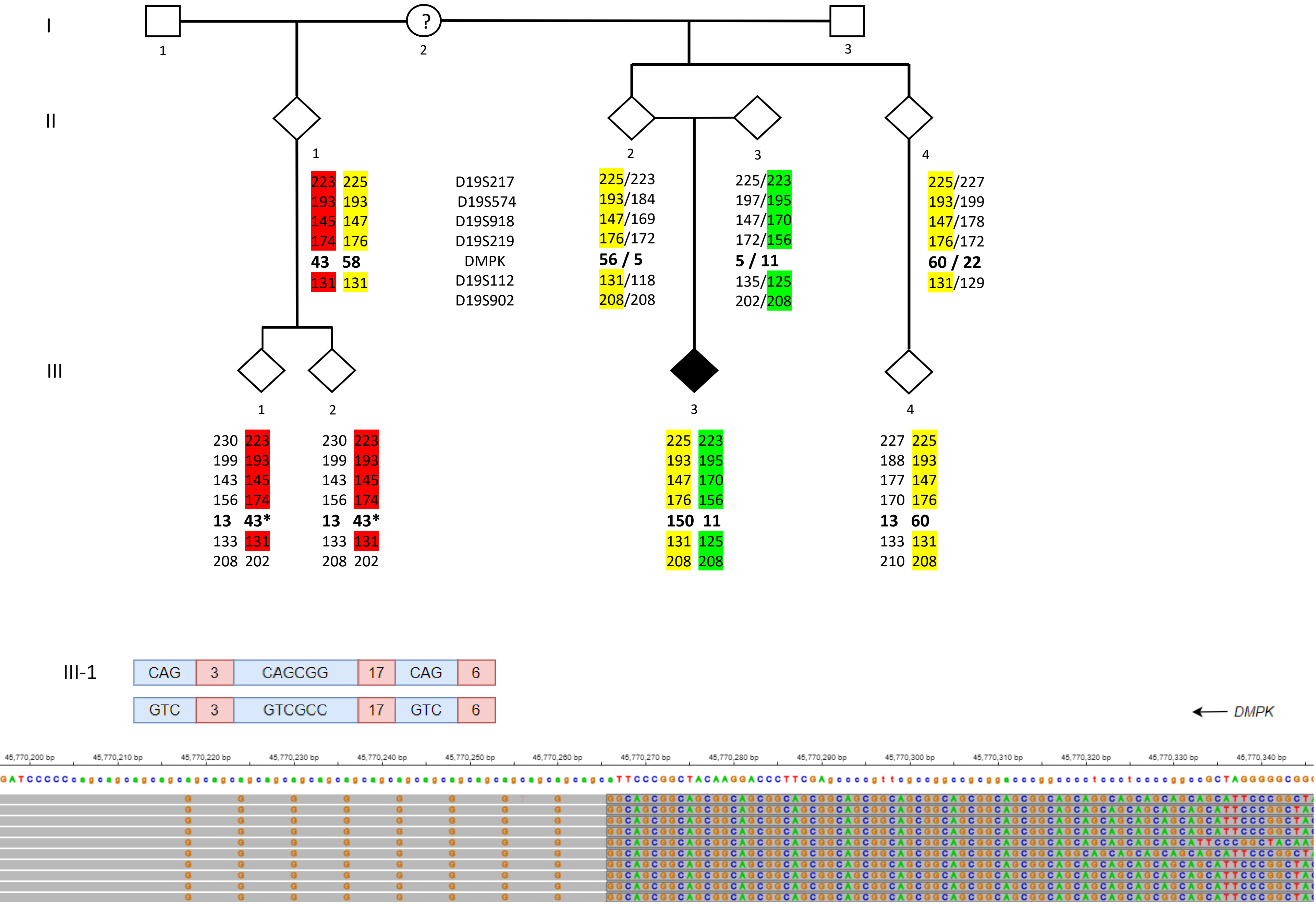

Supplementary fig 4: Family F

III-1

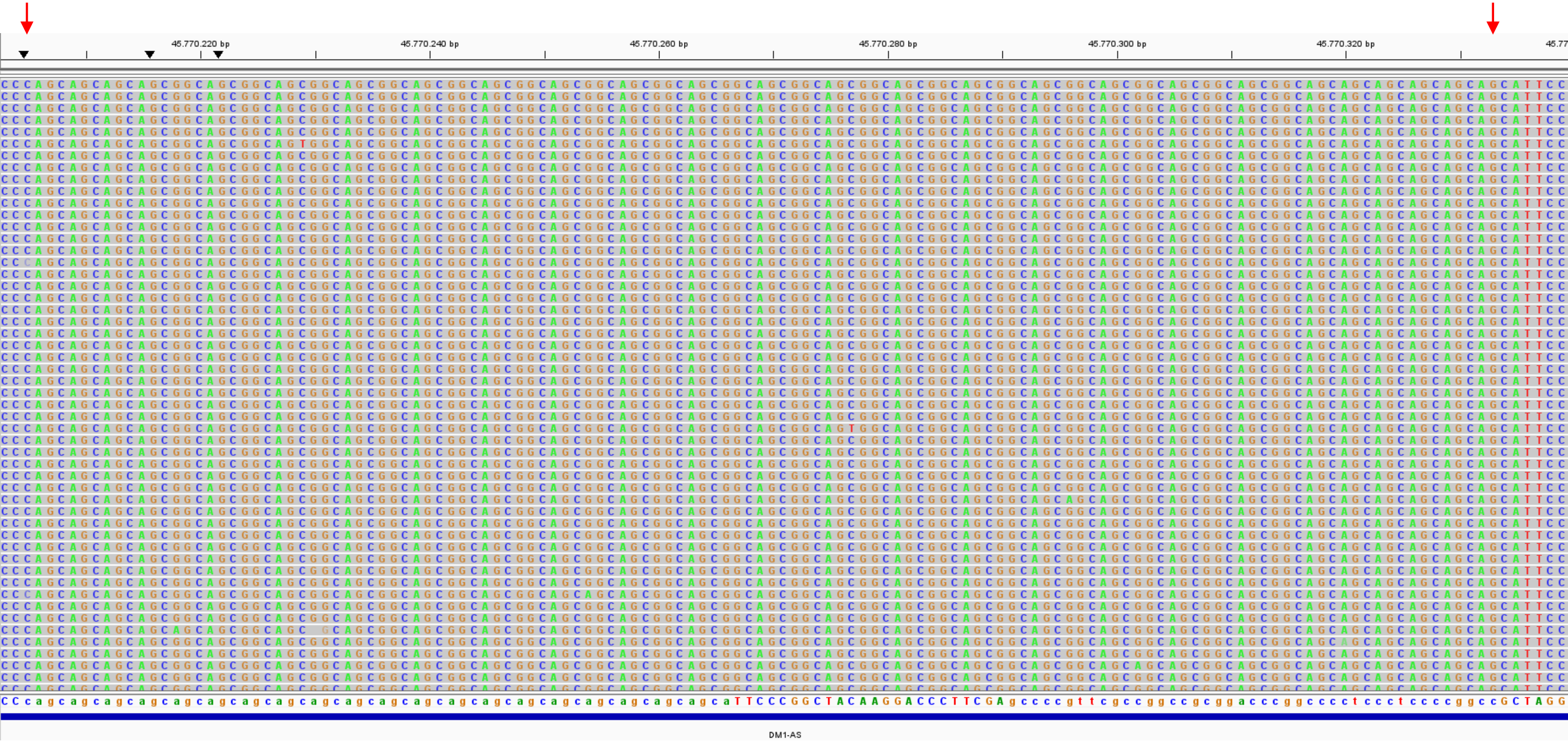

BAM file showing the complete sequence of the DMPK repeat region
