# Supplementary material for: Interrupted CTG repeats in the 37–43 units size range in the 3ʹUTR of DMPK are common alleles
Source: Eur J Hum Genet. 2025 Jul 8;33(11):1547–53. doi: 10.1038/s41431-025-01907-9 (PMC12583562; doi:10.1038/s41431-025-01907-9)
Supplement: Supplementary file 5 — Supplementary fig 5 -Distribution of DMPK CTG repeat alleles [file 41431_2025_1907_MOESM5_ESM.pdf]

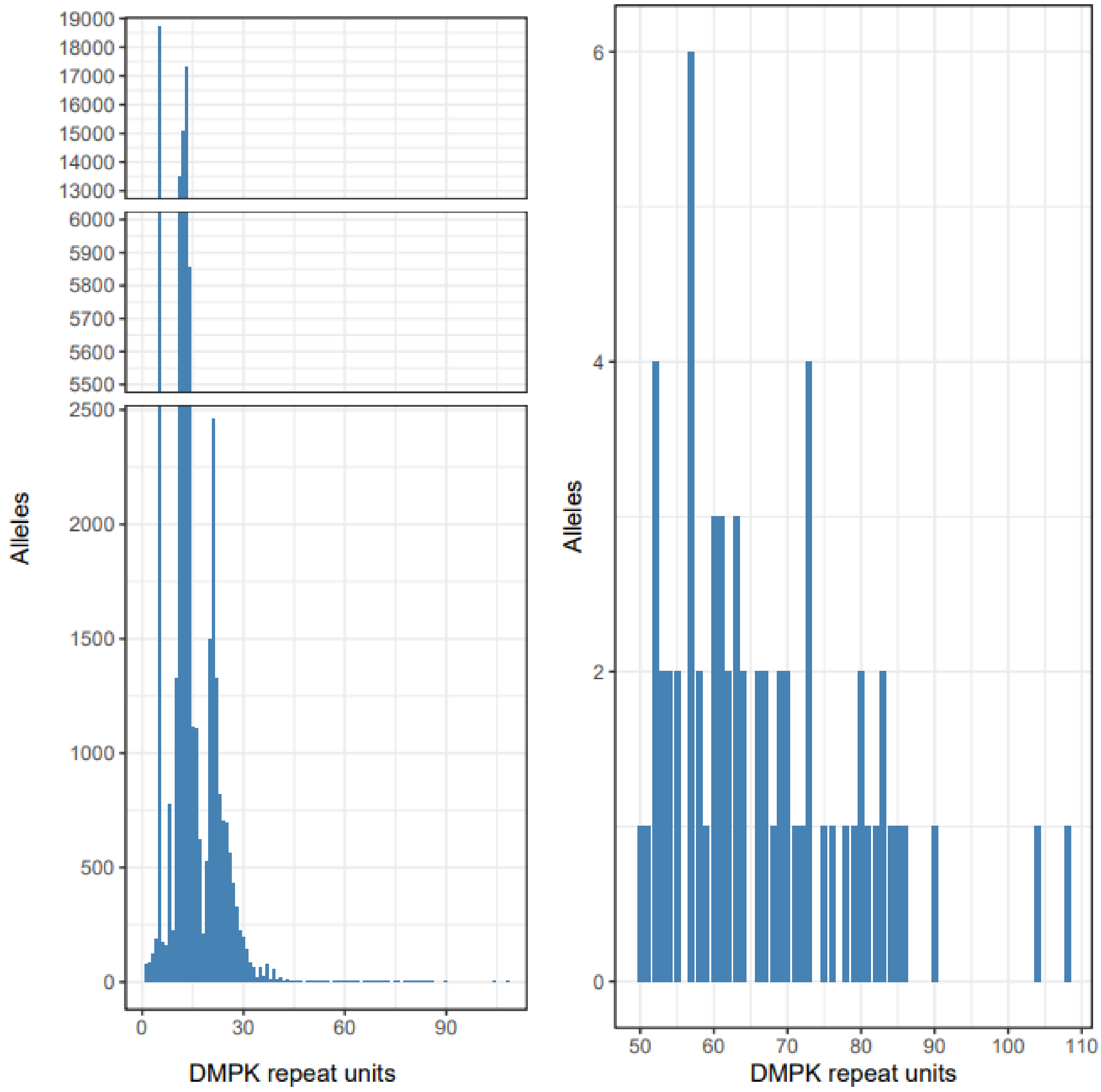

Supplementary fig 5: ***Distribution of DMPK CTG repeat alleles.***

Expansion Hunter output for the DMPK CTG repeat units (DMPK repeat units) was used to create an allele distribution (number of alleles on the Y-axis) plot for the occurence of the different allele sizes in the entire WES cohort of 41,113 samples (82,226 alleles). The Expansion Hunter output was not verified by visual inspection of the realignment plots, and may thus contain genotyping errors.
