# Supplementary material for: Interrupted CTG repeats in the 37–43 units size range in the 3ʹUTR of DMPK are common alleles
Source: Eur J Hum Genet. 2025 Jul 8;33(11):1547–53. doi: 10.1038/s41431-025-01907-9 (PMC12583562; doi:10.1038/s41431-025-01907-9)
Supplement: Supplementary file 6 — supplementary JSON file for CGGCAG detection [file 41431_2025_1907_MOESM6_ESM.docx]

**{**

**"LocusId": "DMPK_3",**

**"LocusStructure": "(CAG)*(CGGCAG)*(CAG)*",**

**"ReferenceRegion": [**

**"chr19:46273462-46273477",**

**"chr19:46273477-46273519",**

**"chr19:46273519-46273534"**

**],**

**"VariantType": [**

**"Repeat",**

**"Repeat",**

**"Repeat"**

**],**

**"VariantId": [**

**"DMPK_3",**

**"DMPK_3_CGGCAG",**

**"DMPK_3_CAG"**

**]**

**}**
